# Development and validation of a predictive model combining radiomics and deep learning features for spread through air spaces in stage T1 non-small cell lung cancer: a multicenter study

**DOI:** 10.3389/fonc.2025.1572720

**Published:** 2025-05-08

**Authors:** Pengliang Xu, Huanming Yu, Wenjian Xing, Shiyu Zhang, Haihua Hu, Wenhui Li, Dan Jia, Shengxu Zhi, Xiuhua Peng

**Affiliations:** ^1^ Department of Thoracic Surgery, The First People’s Hospital of Huzhou, Huzhou, China; ^2^ Department of Radiology, Linghu Hospital, Second Medical Group of Nanxun District, Huzhou, China; ^3^ Department of Radiology, Xishan People’s Hospital of Wuxi, Wuxi, China; ^4^ Department of Radiology, Zhebei Mingzhou Hospital of Huzhou, Huzhou, China; ^5^ Department of Respiratory Medicine, The First People’s Hospital of Huzhou, Huzhou, China; ^6^ Department of Radiology, The First People’s Hospital of Huzhou, Huzhou, China

**Keywords:** deep learning, radiomics, lung adenocarcinoma, artificial intelligence, STAS

## Abstract

**Purpose:**

The goal of this paper is to compare the effectiveness of three deep learning models (2D, 3D, and 2.5D), three radiomics models(INTRA, Peri2mm, and Fusion2mm), and a combined model in predicting the spread through air spaces (STAS) in non-small cell lung cancer (NSCLC) to identify the optimal model for clinical surgery planning.

**Methods:**

We included 480 patients who underwent surgery at four centers between January 2019 and August 2024, dividing them into a training cohort, an internal test cohort, and an external validation cohort. We extracted deep learning features using the ResNet50 algorithm. Least absolute shrinkage selection operator(Lasso) and spearman rank correlation were utilized to choose features. Extreme Gradient Boosting (XGboost) was used to execute deep learning and radiomics. Then, a combination model was developed, integrating both sources of data.

**Result:**

The combined model showed outstanding performance, with an area under the receiver operating characteristic curve (AUC) of 0.927 (95% CI 0.870 - 0.984) in the test set and 0.867 (95% CI 0.819 - 0.915) in the validation set. This model significantly distinguished between high-risk and low-risk patients and demonstrated significant advantages in clinical application.

**Conclusion:**

The combined model is adequate for preoperative prediction of STAS in patients with stage T1 NSCLC, outperforming the other six models in predicting STAS risk.

## Introduction

Lung cancer remains the leading cause of cancer-related deaths worldwide, with non-small cell lung cancer (NSCLC) accounting for approximately 80% of cases (1). Among NSCLC subtypes, lung adenocarcinoma (LUAD) is the most prevalent. Surgical resection is the standard treatment for localized NSCLC, yet postoperative recurrence remains a significant challenge, often linked to unique invasion patterns such as spread through air spaces (STAS) (2, 3). STAS, defined by the WHO in 2015 as the presence of tumor cells in the lung parenchyma surrounding the primary tumor, is recognized as the fourth type of tumor invasion in LUAD and is associated with poorer prognosis (1). For STAS-positive individuals with early-stage lung cancer, lobectomy provides a better clinical prognosis compared to sublobar resection, reducing postoperative tumor recurrence and metastasis (2, 3).

The gold standard for STAS diagnosis is histopathological examination post-surgery. However, preoperative biopsy and intraoperative frozen section examination often face limitations such as low sensitivity and limited tissue samples (4, 5). Consequently, there is a pressing need for non-invasive methods to predict STAS preoperatively, aiding in surgical planning and improving patient outcomes.

Several studies indicate that STAS results in specific radiological features in CT imaging of lung cancer, such as lobulation, vascular convergence, pleural retraction, and the proportion of solid tumor components (6, 7). This suggests that these imaging characteristics may help predict STAS. Radiomics provides a non-invasive method for extracting large amounts of imaging information quickly, generating high-dimensional, mineable data from images, allowing for deeper analysis, prediction, and interpretation of extensive imaging datasets (8). Numerous scholars worldwide have initiated studies on predicting early-stage lung adenocarcinoma STAS using radiomics (9–11). However, traditional radiomics features are manually defined and may not fully capture the tumor phenotype. Deep learning, particularly convolutional neural networks (CNNs), offers a more adaptive approach by extracting features directly from raw images, potentially enhancing predictive accuracy (12–14). Recent studies have demonstrated the effectiveness of deep learning models in predicting STAS, with 3D CNN models showing superior performance (15–17).

The novelty of this study lies in the integration of multiple deep learning models (2D, 2.5D, and 3D) with radiomics models (INTRA, Peri2mm, and Fusion2mm) to predict STAS in stage T1 NSCLC. While previous studies have explored either radiomics or deep learning approaches separately, our combined model leverages the strengths of both methods to improve predictive accuracy. Additionally, this is the first study to introduce a 2.5D CNN model for STAS prediction, which captures features from both the primary tumor slice and adjacent slices, offering a more comprehensive analysis of tumor characteristics. This approach not only enhances the predictive power but also provides a more robust tool for preoperative surgical planning, potentially improving patient outcomes.

## Patients and methods

### Patient characteristics

**Figure 1:**
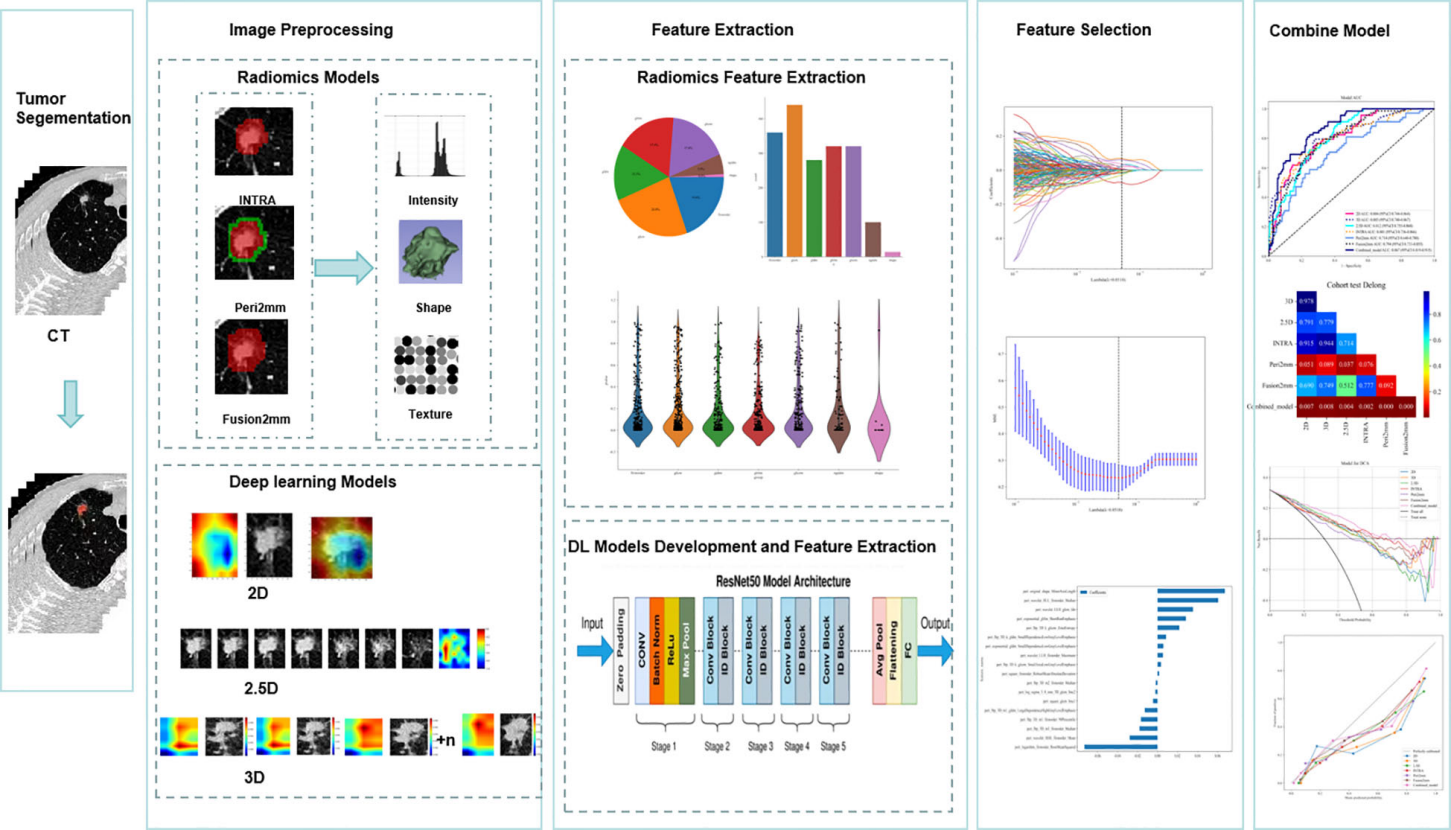
Workflow diagram for the development of the predictive models. Tumor segmentation and region of interest (ROI) delineation are performed by experienced radiologists. The Radiomics model is developed using PyRadiomics. INTRA refers to the internal region of the tumor. Peri2mm represents the region within 2 mm around the tumor. Fusion2mm stands for the combined region of the internal part of the tumor and the region within 2 mm around it. For the deep learning (DL) model, the pre-trained ImageNet ResNet50 is fine-tuned based on our training data. For the two-dimensional deep learning (2D DL) model, the tumor’s maximal ROI cross-section is cropped as the input for ResNet50. For the 2.5D DL model, the tumor’s maximal ROI cross-section and six adjacent CT slices above and below it from seven channels are cropped as the input for ResNet50. For the 3D DL model, The 3D images of the primary tumor, along with their corresponding labels, were then used as input for ResNet50. We applied the Least Absolute Shrinkage and Selection Operator(Lasso) regression technique to select features. The extracted features from four basic models are combined for the early fusion model to train an XGboost classifier. The output probabilities from four basic models are used for the late fusion model to develop a stacking model with an XGboost classifier. Receiver Operating Characteristic (ROC) curves are used to evaluate the predictive model’s performance. The Delong test was employed to compare the AUC values.

All procedures in this research adhered to the guidelines laid out in the Declaration of Helsinki. Approval of the study protocol was granted by the Huzhou First People’s Hospital Ethics Committee, which allowed informed consent to be waived because the study was retrospective. The overall workflow of this study is illustrated in **Figure 1**.

**Figure 2:**
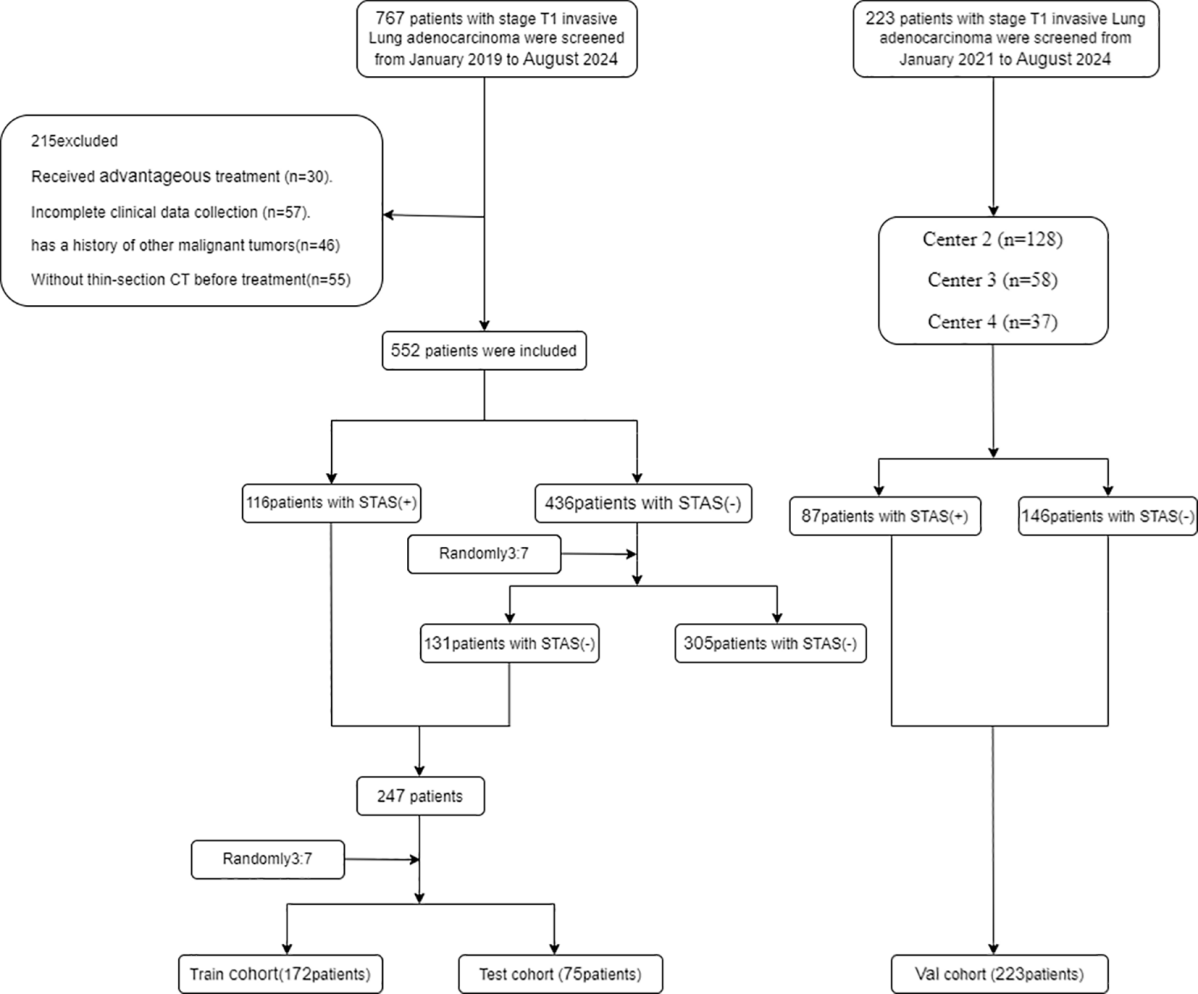
Flowchart diagram shows the patient selection process from four medical centers. STAS indicates spread through air spaces: Val cohort, validation cohort.

We selected 480 patients who underwent a procedure to remove clinical stage IA lung adenocarcinoma (tumor size ≤3 cm) based on the 8th edition of the TNM classification at four centers between January 2019 and August 2024 (**Figure 2**).

The inclusion criteria were: (i) tumor diameter on preoperative CT imaging not exceeding 3 cm according to the 8th edition of TNM staging guidelines; (ii) CT imaging performed within one month before surgery; (iii) pathological confirmation of invasive lung adenocarcinoma.

Exclusion Criteria were: (i)History of neoadjuvant therapy; (ii)Presence of multiple pulmonary nodules on preoperative CT scans; (iii)Past or present diagnosis of other malignant tumors; (iv)pure ground glass nodules on CT; (v)Evidence of distant metastasis.

The development dataset included 247 lesions obtained from 480 patients. We partitioned the dataset at random into a training set of 172 lesions and a test set of 75 lesions, maintaining a 7:3 ratio. Approximately 30% of the lesions in each dataset tested positive for STAS. To validate the models further, we included 233 individuals whose lung cancer was surgically resectioned during its early stages at three institutions between January 2019 and August 2024 as the validation set. Thus, the outcome cohort for predicting STAS consisted of 480 patients from the training, test, and internal validation sets.

### Histopathological evaluation

Clinical outcomes involved re-evaluating the hematoxylin and eosin tissue sections using WHO criteria for STAS to establish an agreement on STAS status. Tum cells in lung air spaces identified STAS positivity away from the primary tumor’s boundary. There are three main forms of STAS: (i) air spaces filled with micro nipple structures that do not have central fibrovascular cores; (ii) solid nests where air spaces are filled with the tumor’s solid components; and (iii) air spaces with multiple discrete and separate single cells.

### Clinical variables

Patient clinical information was gathered from our hospital’s electronic medical records, including gender, age, smoking history, and levels of CEA and CA125.

### Image acquisition and preprocessing

All patients received a standard chest CT scan. Comprehensive details regarding treatment protocols and clinical information are outlined in the **Supplementary Methods**. Initially, we resampled all the CT images and standardized the voxel dimensions to 1 mm x 1 mm x 1 mm. Subsequently, we standardized the window width and window level to 1300 and -300, respectively, which we determined to be suitable for the segmentation of the region of interest (ROI).

### Region of interest segmentation

A radiologist with forty-five months of expertise in chest CT manually sketched the ROI around the tumor outline, referred to as INTRA. They segmented each slice of the images in the lung window (width: 1300 HU; level: -300 HU) using the open-source software ITK-SNAP (version 3.8.0, http://www.itksnap.org). All ROIs that radiologist A had manually segmented were reviewed by radiologist B, who has ten years of experience in the field. We expanded the initial ROIs by 2 mm into the peritumoral regions, which we termed the peri2mm areas. We then combined the tumor ROIs with the peri2mm areas to create a new region of interest called the fusion2mm ROI. We utilized this data to perform an intraclass correlation coefficient (ICC) analysis, assessing the dependability and uniformity of the ROI delineations.

### Radiomics feature extraction and model construction

Our study used PyRadiomics to extract the radiomics features from INTRA, Peri2mm, and Fusion2mm. We extracted a total of 1,834 radiomic features, such as first-order, shape-based, and texture features; detailed parameters for this extraction can be found on the PyRadiomics website (https://pyradiomics.readthedocs.io/en/latest/).

Radiomic features were standardized using z-score normalization. To handle the high degree of feature-to-feature correlation (Spearman correlation coefficient = 0.9 or higher), we used a greedy, recursive filtration technique. This method iteratively removes the most redundant features until no features with a correlation coefficient above 0.9 are left. Subsequently, features that demonstrated high stability were retained, as indicated by intraobserver and interobserver ICC values greater than 0.75.

To enhance the selection of features, we applied the multivariate Least Absolute Shrinkage and Selection Operator(Lasso) regression technique. Extreme Gradient Boosting (XGBoost) is a machine learning technique for solving classification and regression predictive modeling challenges. For precise gradient boosting implementation, T. Chen and C. Guestrin (18) created it. For accurate results and to avoid over-fitting during training, it uses sequentially built shallow decision trees. We trained an XGboost classifier to develop the predictive models. A five-fold cross-validation method was adopted to determine the most suitable model configuration and hyperparameter adjustments. The XGboost classifier was fed a training dataset, where each entry included a feature set and a label indicating whether STAS was present. The outcome was a classifier model foretrained to estimate the probability of STAS in patients within the test and validation groups. This model’s output gave a probability score (ranging from 0 to 1) indicating the presence of STAS in the evaluated patient.

### 2D model development and feature extraction

In 2D deep learning applications, the bounding box is the rectangular boundary of the most significant tumor cross-section. This box is employed to crop the maximum tumor ROI. The cropped ROI is then saved in PNG format. The ResNet50 model was first pre-trained on the ImageNet dataset, and then transfer learning was used on the training set. ImageNet is a large database that contains millions of labeled images that are organized into categories. Transfer learning based on ImageNet has been used in many medical studies. We used a global fine-tuning strategy to update the model parameters, which improved the ResNet50’s ability to predict STAS.

The images of primary tumors and their corresponding labels were utilized as inputs for the 2D Convolutional Neural Network (2D CNN). In the beginning stages of training, the parameters of ResNet50 were iteratively updated through backpropagation, and the cross-entropy loss function was employed to interpret the output probabilities and pathological labels. The learning rate was set at 1×10^-4, and the parameters were updated using the Adam optimizer. The following batch size was used: 64, along with L2 regularization and early stopping strategies to avoid overfitting. After completing the training of ResNet50, we used it to extract 2048 deep learning features from the ROI image using the penultimate average pooling layer of ResNet50.

### 2.5D model development and feature extraction

Considering that 2D CNN only extracts features from the most significant ROI slice, lacking features from adjacent slices, we introduced a 2.5D CNN for feature extraction. In addition, it includes the most essential ROI slice and encompasses three adjacent axial slices above and below it. In contrast to the approach in this study (19), where CNNs are trained separately for each layer of image slices, and their predicted features are later fused, our goal is to simplify training by using seven-channel images together.

The presence of STAS was indicated by assigning identical labels to multiple image patches from the same patient. The images of primary tumors and their corresponding labels from seven channels were utilized as inputs for the 2.5D CNN. After training ResNet50, by utilizing the penultimate average pooling layer, we were able to extract 2048 deep learning features from every patch.

### 3D model development and feature extraction

In 3D deep learning, the bounding box refers to the smallest enclosing cube of the tumor ROI. Next, the 3D CNN was fed the primary tumor’s 3D pictures along with their labels. With the help of backpropagation and the Adam optimizer, the network parameters were fine-tuned. We carried out 100 training epochs with a learning rate set to 0.02. Ultimately, the trained 3D CNN could predict the patient’s STAS. Two thousand forty-eight features of the tumor image were extracted from the penultimate averaging pooling layer of the 3D ResNet50 model as the 3D deep learning features for each patient.

### Construction of the combined model

Merging the output probabilities from multiple models is known as late fusion or decision-level fusion. The six underlying models’ output probabilities were combined using a stacking ensemble method. A five-fold cross-validation technique was used on the training set to establish the optimal hyperparameters for the XGboost classifier. The final result was testing on both internal and external datasets to determine the performance of the “combined model,” the stacking model that had been trained optimally.

### Statistical analysis

We employed Chi-square or Fisher’s tests for the comparison of categorical variables and utilized the Mann–Whitney U or independent T-test for continuous variables. The effectiveness of the predictive model was evaluated using Receiver Operating Characteristic (ROC) curves, AUC, accuracy, sensitivity, and specificity. To calculate the 95% Confidence Interval (CI) for AUC, the ci. Auc function from the pROC package in R was used. The Delong test was adopted to compare the AUC values. A p-value below 0.05 was deemed to indicate statistical significance. The statistical analysis was conducted in R(version 4.4.1) and the scikit-learn package (version 0.18) in Python 3.13.

## Results

### Baseline characteristics of the patients

This study comprised 480 individuals with clinical T1 stage invasive lung adenocarcinoma, comprising 172 patients in the training set (83 STAS-positive and 89 STAS-negative), 75 patients in the internal test set (33 STAS-positive and 42 STAS-negative), and 233 patients in the external validation set (146 STAS -positive and 87 STAS -negative). Patient clinical data, CT characteristics, and pathological information were recorded. In **Table 1** you can see all of the patients’ clinical baseline data. In univariate analysis, smoking and preoperative CEA level are risk factors for STAS, while multivariate analysis suggests that CEA level is an independent risk factor for STAS (**Table 2**).

**Table 1:** Baseline characteristics of study sets.

Variable	Training set (n = 172)	Test set (n = 75)	External validation set (n = 233)	P value
Age	65.058 ± 9.332	65.800 ± 9.317	64.021 ± 10.991	0.347
Gender				0.564
Male	88 (51.163)	35 (46.667)	126 (54.077)	
Female	84 (48.837)	40 (53.333)	107 (45.923)	
Somke				0.805
No	132 (76.744)	59 (78.667)	175 (75.107)	
Yes	40 (23.256)	16 (21.333)	58 (24.893)	
CEA				0.556
Negative	141 (81.977)	57 (76.000)	186 (79.828)	
Positive	31 (18.023)	18 (24.000)	47 (20.172)	
CA125				0.758
Negative	166 (96.512)	71 (94.667)	222 (95.279)	
Positive	6 (3.488)	4 (5.333)	11 (4.721)	

**Table 2:** Logistic regression analysis identified independent clinical predictors for STAS (+).

Variable	Univariable logistic regression		Multivariable logistic regression	
	OR(95%CI)	*P* value	OR(95%CI)	*P* value
Age	0.999(0.995,1.003)	0.731		
Gender	1.066(0.910,1.249)	0.503		
Somke	2.636(1.473,4.721)	0.006	2.011(1.097,3.684)	0.058
CEA	5.200(2.328,11.612)	0.001	4.299(1.895,9.757)	0.003
CA125	0.500(0.120,2.077)	0.423		

### Detailed analysis of radiomics, 2D,2.5D and 3D deep learning features

By applying LASSO feature selection, we identified 31, 18, and 29 critical radiomic features from the INTRA, Peri2mm, and Fusion2mm datasets out of 1834 radiomic features. In addition, 28 2D deep learning (DL) features, 48 2.5D DL features and eight 3D DL features were identified as having notably high weights, qualifying them as the most significant features (**Supplementary Figure S1**). We utilized T-SNE for dimension reduction to visualize these features (**Supplementary Figure S2**). As illustrated in **Supplementary Figure S3**, 2.5D DL features demonstrated superior prediction accuracy compared to radionics, 3D, and 2D DL features. From **Supplementary Figure S4**, it can be seen that 2.5D has a high degree of discrimination.

### Performance analysis of the radiomics models, deep learning models, and the combined model

**Figure 3:**
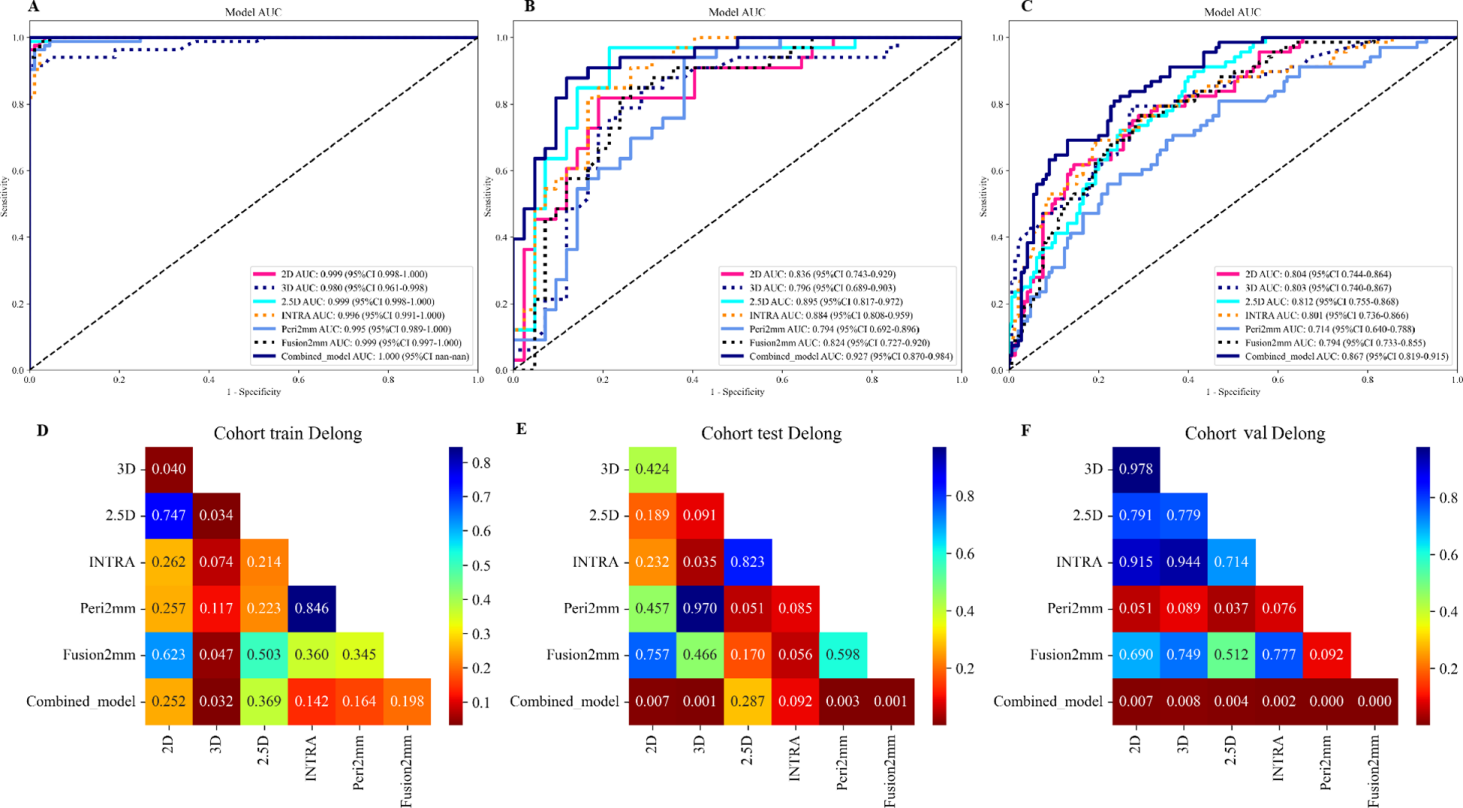
Performances for spread through air spaces (STAS) prediction. The receiver operating characteristic (ROC) curves of the 2D DL model, 3D DL model, 2.5D DL model, INTRA model, Peri2mm model, Fusion2mm model, and combined model in the training set **(A)**, internal test set **(B)**, and external validation set **(C)**. AUC indicates the area under the curve. P value was calculated through the Delong test. The comparison of the area under the AUC curves among various models in the training set **(D)**, internal test set **(E)**, and external validation set **(F)**.

**Figure 4:**
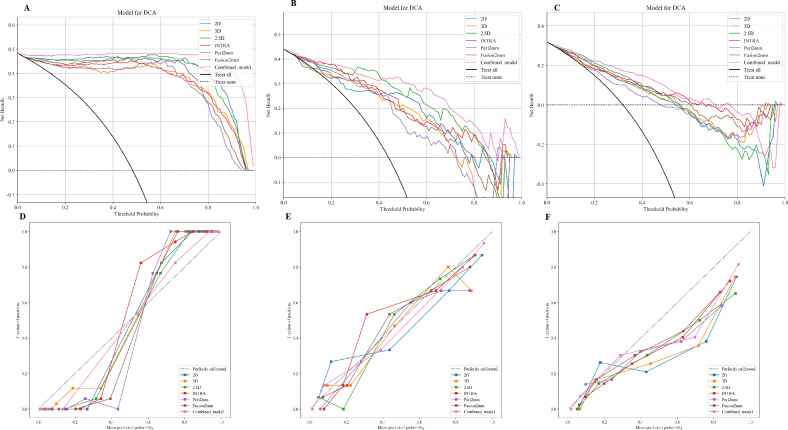
Calibration curves of the 2D DL model, 3D DL model, 2.5D DL model, INTRA model, Peri2mm model, Fusion2mm model, and combined model in the training set **(A)**, internal test set **(B)**, and external validation set **(C)**. The decision-curve analysis plot depicts the standardized net benefit of each model in the training set **(D)**, internal test set **(E)**, and external validation set **(F)**.

**Table 3** presents the diagnostic indicators for the predictive models used within the study sets. The combined model reached the greatest AUC, which is between 0.867 and 1.000, showing significant superiority over all models in the validation set (P = 0.000-0.007) (**Figure 3**). In contrast to the 2.5D model(AUC=0.999), the AUCs for the 3D DL, 2D DL, INTRA, Peri2mm, and Fusion2mm were 0.980 (P=0.034), 0.999(P =0.747), 0.996 (P =0.214), 0.995(P =0.223), and 0.999 (P =0.503)respectively in the training set. Plus, the 2.5D DL model achieved an impressive AUC of 0.895 on the test set and 0.812 on the validation set, indicating its satisfactory performance. However, the test and validation sets found no significant differences in AUC between the 2.5D DL model and the 2D DL, 3D DL, and radiomics models (**Figure 3**). **Figure 4** shows that the combined model’s calibration curves were very consistent over a wider range of probabilities in the data sets. The DCA curves showed that the combined model was more beneficial overall (**Figure 4**).

**Table 3:** Performances of the predictive models in the study sets.

Model	Accuracy	AUC	95% CI	Sensitivity	Specificity	Precision	Threshold
Training set (n = 172)							
2D	0.977	0.999	0.9978 - 1.0000	0.988	0.966	0.965	0.428
3D	0.953	0.980	0.9613 - 0.9983	0.904	1.000	1.000	0.563
2.5D	0.988	0.999	0.9983 - 1.0000	0.976	1.000	1.000	0.588
INTRA	0.977	0.996	0.9911 - 1.0000	0.976	0.978	0.976	0.461
Peri2mm	0.971	0.995	0.9889 - 1.0000	0.952	0.989	0.987	0.613
Fusion2mm	0.977	0.999	0.9966 - 1.0000	0.976	0.978	0.976	0.549
Combined_model	0.994	1.000	1.0000 - 1.0000	0.988	1.000	1.000	0.672
Test set (n = 75)							
2D	0.800	0.836	0.7435 - 0.9290	0.788	0.810	0.765	0.574
3D	0.760	0.796	0.6894 - 0.9029	0.818	0.714	0.692	0.395
2.5D	0.853	0.895	0.8169 - 0.9724	0.939	0.786	0.775	0.391
INTRA	0.813	0.884	0.8082 - 0.9595	0.818	0.810	0.771	0.313
Peri2mm	0.733	0.794	0.6918 - 0.8955	0.879	0.619	0.644	0.443
Fusion2mm	0.773	0.824	0.7268 - 0.9204	0.818	0.738	0.711	0.428
Combined_model	0.867	0.927	0.8703 - 0.9839	0.848	0.881	0.848	0.499
External validation set (n = 233)							
2D	0.723	0.804	0.7440 - 0.8642	0.750	0.710	0.548	0.527
3D	0.742	0.803	0.7399 - 0.8665	0.779	0.724	0.570	0.523
2.5D	0.681	0.812	0.7555 - 0.8683	0.882	0.586	0.500	0.317
INTRA	0.765	0.801	0.7363 - 0.8656	0.676	0.807	0.622	0.561
Peri2mm	0.653	0.714	0.6404 - 0.7882	0.691	0.634	0.470	0.425
Fusion2mm	0.723	0.794	0.7325 - 0.8550	0.750	0.710	0.548	0.433
Combined_model	0.770	0.867	0.8192 - 0.9151	0.809	0.752	0.604	0.423

## Discussion

STAS serves as a crucial risk factor for an unfavorable postoperative prognosis of stage T1 stage lung adenocarcinoma(LUAD) (20, 21). An earlier study found that the STAS-positive group had a higher proportion of solid-based and micropapillary-based types in the postoperative pathological results (22). When evaluating the aggressiveness of early-stage lung adenocarcinoma, STAS is a crucial indicator to consider. This finding is in line with earlier research (23, 24) showing that T1 stage LUAD with STAS is more malignant. Existing literature evaluated STAS using various morphological parameters of pulmonary nodules through routine preoperative imaging (25, 26). These parameters included the proportion of solid components, the presence of abnormal bronchial gas phase, the largest diameter of the lesion, and the blurred ground-glass boundaries around subsolid lesions (27, 28). This novel method is highly dependent on the radiologist’s practical expertise. Thus, it is essential to thoroughly investigate the imaging features of T1 stage LUAD to develop a predictive model that offers greater diagnostic efficiency and more precise diagnostic criteria.

To the best of our knowledge, this study is the initial one concentrating on the 2.5D DL model in predicting STAS of LUAD. Radiomic features from intratumoral regions are widely used to indicate the prognosis of LUAD (29–31). But there has not been much research on using peritumoral imaging features to help with these predictions, and the peritumoral region’s definition is still up for debate. STAS typically occurs around 2-3mm from the tumor (32, 33). Consequently, this study treats the 2 mm peri-tumoral area as a separate Region of Interest (ROI) for extracting radiomic features.

Additionally, we integrated the tumor and the peri-tumoral 2mm for radiomics feature selection to predict STAS. Then, we contrasted the performance of 2D, 2.5D, and 3D DL models with radiomic models, including the INTRA model, Peri2mm model, and Fusion2mm model. Ultimately, we developed a joint model that combines 2D CNN, 2.5D CNN, and 3D CNN deep learning models with the INTRA, Peri2mm, and Fusion2mm radiomic models to improve the prediction of STAS status. Clinical data analysis identifies smoking and preoperative serum CEA levels of 5μg/L or higher as risk factors for STAS. Multifactorial logistic regression analysis indicates that preoperative serum CEA levels are independent predictive factors for STAS. These findings align with the conclusions of previous studies (34).

In this study, the AUC values for the radiomics models are INTRA at 0.996, Peri2mm at 0.995, and Fusion2mm at 0.999 in the training set. In the test set, they are 0.884, 0.794, and 0.824, respectively. Furthermore, during external validation, the AUC values for the models were 0.801 for INTRA, 0.714 for Peri2mm, and 0.794 for Fusion2mm. In the radionics model, INTRA emerged as the best-performing model in the test set, exhibiting a higher AUC than the Fusion2mm and Peri2mm models, with statistically significant differences. The same conclusion was consistent in the external validation set. From the INTRA model, we extracted eleven first-order features, one shape feature, and nineteen second-order indexes, which include GLCM, GLDM, GLRLM, GLSZM, and NGTDM features. Eight First-order features, one Shape feature, and nine second-order indexes, including GLCM, GLDM, GLRLM, and GLSZM features, were extracted from the Peri2mm. Eight First-order features and twenty-one second-order indexes, including GLCM, GLDM, GLRLM, GLSZM, and NGTDM features, were extracted from the Fusion2mm.

First-order statistics refer to voxel intensity distribution in the image area defined by the mask using basic metrics. Meanwhile, second-order parameters relate to the spatial relationships of voxel intensity (34). Shape describes the geometry of the region of interest. It includes volume, sphericity, surface area, and compactness, which assist in tumor diagnosis, evaluate treatment effects, and facilitate research data comparisons. Therefore, various gray-level features indicating intratumor heterogeneity were included in the radiomics model, proposing their contribution to the observed high diagnostic accuracy (35, 36).This may be related to the internal differences within the tumor. Studies (37) have shown that after incorporating semiquantitative analysis of SUV ratios, especially the SUVmax nodule/SUVmean BP ratio, the specificity of this imaging modality in patients can be significantly improved.

Additionally, our results showed that the first-order_Maximum feature is strongly associated with STAS, having the highest estimated coefficient among the selected first-order parameters from the INTRA ROI. The first-order_Maximum feature refers to the maximum value among the gray values of all voxels within the region of interest (ROI) (38). In addition, first-order_Root Mean Squared was the highest estimate coefficient among the selected first-order parameters from Peri2mm ROI. In tumor images, factors such as the tumor tissue’s internal cell density and metabolic state can change the gray values. “First-order_Root Mean Squared” can reflect the overall magnitude of these changes in gray values. A higher root mean squared value indicates more significant fluctuations in the gray values within the region of interest (ROI); the tissue has a higher heterogeneity, which may imply multiple different cell components or physiological states within the tumor tissue.

Similar to how Size-zone non-uniformity and level variance, two radionics features that represent gray-level characteristics, have been linked to STAS-positive tumors in earlier studies (36, 38). Furthermore, our clinical-CT model demonstrated that the solid-density type and lower GGO ratio were the most important determinants of STAS risk. The features extracted by the radiomics analysis are confirmed to be reliable and interpretable, as computer-automated feature extraction is more objective and accurate than subjective and manual measurements.

ResNet50, a popular CNN, is commonly used in medical image recognition and semantic segmentation (39, 40). ResNet, as a deep residual network, is notable for its “skip connections,” which add cross-layer links in each residual block (41). This enables direct information transfer to the following convolutional layers, preserving the original features and preventing their gradual loss. Therefore, ResNet provides distinct advantages in feature extraction over other CNN architectures. Numerous studies in radiomics have leveraged ResNet for this purpose, demonstrating its established effectiveness in medical image feature extraction (42–44). For our analysis, we employed a ResNet50 model pre-trained on ImageNet (45, 46), a comprehensive dataset in computer vision, to derive deep learning features.

In the deep learning models, the 2.5D deep learning model was the top performer in the test set, achieving a higher AUC (0.895) compared to the 2D(AUC=0.836) and 3D(AUC=0.796) models. However, the differences were not statistically significant. The external validation set produced similar results, confirming the findings from the test set. Among all the radiomics and deep learning models evaluated, the 2.5D deep learning model stood out as the best-performing single model. Meanwhile, the INTRA model significantly outperformed the 2D and 3D deep learning models. The findings reveal that these deep learning features only sometimes enhance prediction accuracy compared to traditional radiomics features extracted using PyRadiomics.

In the test set, the INTRA model that used only traditional radiomics features performed better than the 3D deep learning model. This finding is backed by research conducted by Feng et al. (47), which found that models using deep learning features from VGG19 had lower AUC scores than those using other non-machine learning techniques. In a multicenter cohort study by Cui et al. (48), deep learning and manually crafted radiomics features were used to develop a nomogram predicting the response to neoadjuvant chemotherapy in advanced gastric cancer. However, these studies and ours noted that models combining deep learning with radiomics generally outperformed standalone models. The models developed using deep learning features did not outperform those built with conventional handcrafted radiomics features; however, models that incorporated deep learning features demonstrated enhanced performance.

Nevertheless, our research also has certain drawbacks. Firstly, considering the long follow-up duration, this research was retrospective. Secondly, the relatively small sample size may affect the generalizability of the results. And the relatively small sample size may increase the risk of overfitting, particularly in complex models like deep learning. However, the model exhibited good performance in the external validation set, somewhat alleviating this shortcoming. Third, the samples are limited in terms of their demographics and ethnicities, so it will be necessary to verify the results in the future using samples from multiple ethnic groups. In the future, we will concentrate on improving and validating the combined model by conducting high-quality, multicenter prospective studies.

## Conclusion

To sum up, this retrospective cohort study presents a novel model that combines preoperative CT-based radiomics and deep learning with postoperative pathology-confirmed adenocarcinoma spread to predict postoperative metastasis in stage I lung adenocarcinoma. This model demonstrated superior predictive efficacy in internal and external validation sets, suggesting it can help formulate surgical and postoperative treatment strategies for patients with stage T1 lung adenocarcinoma.

## Data Availability

The raw data supporting the conclusions of this article will be made available by the authors, without undue reservation.
