# Supplementary material for: Development and validation of a predictive model combining radiomics and deep learning features for spread through air spaces in stage T1 non-small cell lung cancer: a multicenter study
Source: Front Oncol. 2025 May 8;15:1572720. doi: 10.3389/fonc.2025.1572720 (PMC12094994; doi:10.3389/fonc.2025.1572720)

**Supplementary materials**

Development and validation of a predictive model combining clinical, radiomics, and deep learning features for Spread Through Air Spaces in Stage T1 Non-Small Cell Lung Cancer:a multicenter study

**Supplementary Methods**

1. **CT parameters of different Centres**

The First People’s Hospital of Huzhou：

CT scancer: German Siemens Definition AS 64-slice 128-slice spiral CT. The scanning range extended from the thoracic inlet to the diaphragm. The subject was placed in a supine position, then instructed to inhale deeply and hold their breath. The scanning parameters were as follows: tube voltage of 120 kV, tube current of 120 mA, window width of 1300-1500, window level of -600 to -700, pitch of 1.0, and rack rotation time of 0.33 seconds per 360 degrees. The lung window was reconstructed with the lung method, with a reconstruction thickness and slice spacing of 1.25 mm. The mediastinal window reconstruction thickness and slice spacing were 5 mm.

Zhebei Mingzhou Hospital of Huzhou:

CT scancer: Siemens Somatom Force dual source CT machine, instruct the patient to lie on their back, head first, and perform a chest scan; Parameters: Tube voltage 120kV, tube current 60-120mA, layer thickness 5-8mm, matrix 512 × 512; Reconstruct the image with a layer thickness of 0.625-2.5mm, set the window as a lung window, with a width of 1500HU and a height of -500HU.

Second Medical Group of Nanxun District:

CT scancer: Philips Brilliance 16 slice spiral CT or Philips Brilliance CT 64 slice spiral CT is used for chest CT plain scan or enhanced examination. The detector line width is 16 × 1.50mm, the tube voltage is 120kV, and the tube current is automatically adjusted; After scanning, thin layer image reconstruction is performed with a layer thickness of 2mm and a layer spacing of 1mm.

Xishan People's Hospital of Wuxi:

CT scancer: Discovery 750,64-slice CT scanner (GE Healthcare, Milwaukee, USA)

and a 16-slice CT scanner (Brightspeed, GE Healthcare, Milwaukee, USA) using the following parameters: 120 kVp tube voltage, 100-360 mA or 10-400 mA tube current (using automatic tube current modulation technique), 64 × 0.625 mm or 16 × 0.625 mm detector collimation, a matrix of 512 × 512, a pitch of 0.984:1 or 1.375:1, a gantry rotation time of 0.6 s, and a slice thickness of 5 mm. Patients held their breath for scanning in the supine position. The scanning area covered the whole chest. Axial images were reconstructed with a slice thickness of 1.25 mm and an interval of 1.0 mm with a lung algorithm.

1. **Radiomics feature extraction**

This study utilized the majority of default parameter settings provided by the PyRadiomics package. A fixed bin number of 64 was used for image discretization. The aggregation method for feature extraction can be found in Supplementary Table S1. We applied feature transformation using eight different filters, namely LoG (Laplacian of Gaussian), Wavelet, LBP3D (Local Binary Patterns in 3D), Exponential, Square, SquareRoot, Logarithm, and Gradient. These filters help in extracting additional information from the radiological images.

**3. The interpretation of parameters in convolutional neural networks**

Learning Rate: In a convolutional neural network (CNN), the learning rate is a hyperparameter that determines the step size at which the model’s parameters are updated during the training process. It controls the speed and magnitude of the parameter updates, influencing how quickly the model adapts to the training data.

L2 Regularization: L2 regularization, also known as weight decay, is a technique used in CNNs to prevent overfitting. It involves adding a regularization term to the loss function that penalizes large parameter values. This regularization term encourages the model to have smaller parameter values, effectively simplifying the model and reducing its sensitivity to individual data points. By including L2 regularization, the CNN becomes more robust and generalizes better to unseen data.

Batch Size: In CNNs, the training data is divided into smaller batches during the training process.

The batch size refers to the number of samples that are processed together before updating the model’s parameters. When training with a larger batch size, more samples are processed simultaneously, which can lead to faster training as it takes advantage of parallelization. However, larger batch sizes may require more memory and can result in slower convergence or poorer generalization. Conversely, smaller batch sizes may take longer to train but can result in better convergence and generalization as they offer more frequent parameter updates and increased model variability.

**4 .Radiomic features from the INTRA, Peri2mm, and Fusion2mm**

INTRA:

intra_exponential_glcm_Inverse Variance

intra_exponential_glszm_Small Area High Gray Level Emphasis,

intra_exponential_glszm_Zone Percentage,

intra_lbp_3D_k_glrlm_Short Run Low Gray Level Emphasis,

intra_lbp_3D_k_glszm_Gray Level Variance,

intra_lbp_3D_k_ngtdm_Contrast

intra_lbp_3D_m1_glcm_Inverse Variance,

intra_lbp_3D_m2_gldm_Large Dependence Low Gray Level Emphasis,

intra_lbp_3D_m2_glszm_Gray Level Non Uniformity Normalized,

intra_log_sigma_2_0_mm_3D_firstorder_Kurtosis,

intra_logarithm_firstorder_Maximum,

intra_logarithm_glszm_Small Area Emphasis,

intra_logarithm_glszm_Zone Percentage,

intra_original_firstorder_Kurtosis,

intra_original_glcm_Cluster Shade,

intra_original_shape_Elongation,

intra_square_firstorder_Median,

intra_square_glcm_Cluster Shade,

intra_square_glcm_Inverse Variance,

intra_wavelet_HLH_firstorder_Kurtosis,

intra_wavelet_HLL_glcm_Cluster Prominence,

intra_wavelet_HLL_glcm_Difference Variance,

intra_wavelet_LHL_firstorder_Maximum,

intra_wavelet_LHL_firstorder_Mean,

intra_wavelet_LHL_glcm_Idn,

intra_wavelet_LLH_firstorder_10Percentile,

intra_wavelet_LLH_firstorder_Median,

intra_wavelet_LLH_firstorder_Range,

intra_wavelet_LLH_glcm_Idm,

intra_wavelet_LLH_ngtdm_Coarseness,

intra_wavelet_LLL_firstorder_Minimum

Peri2mm:

peri_exponential_gldm_Small Dependence Low Gray Level Emphasis,

peri_exponential_glrlm_Short Run Emphasis,

peri_lbp_3D_k_gldm_Small Dependence Low Gray Level Emphasis,

peri_lbp_3D_k_glszm_Small Area Low Gray Level Emphasis,

peri_lbp_3D_k_glszm_Zone Entropy,

peri_lbp_3D_m1_firstorder_90Percentile,

peri_lbp_3D_m1_firstorder_Median,

peri_lbp_3D_m1_gldm_Large Dependence High Gray Level Emphasis,

peri_lbp_3D_m2_firstorder_Median,

peri_log_sigma_3_0_mm_3D_glcm_Imc2,

peri_logarithm_firstorder_Root Mean Squared,

peri_original_shape_Minor Axis Length,

peri_square_firstorder_Robust Mean Absolute Deviation,

peri_square_glcm_Imc1,

peri_wavelet_HHL_firstorder_Mean,

peri_wavelet_HLL_firstorder_Median,

peri_wavelet_LLH_firstorder_Maximum,

peri_wavelet_LLH_glcm_Idn

Fusion2mm:

fusion_exponential_glcm_Inverse Variance,

fusion_exponential_gldm_Dependence Non Uniformity Normalized,

fusion_exponential_gldm_Small Dependence High Gray Level Emphasis,

fusion_exponential_glszm_Small Area High Gray Level Emphasis,

fusion_exponential_glszm_Zone Percentage,

fusion_lbp_3D_k_glrlm_Short Run Low Gray Level Emphasis,

fusion_lbp_3D_m1_gldm_Large Dependence Low Gray Level Emphasis,

fusion_lbp_3D_m1_glszm_Zone Entropy,

fusion_lbp_3D_m2_firstorder_Maximum,

fusion_lbp_3D_m2_glszm_Gray Level Variance,

fusion_lbp_3D_m2_glszm_Small Area Emphasis,

fusion_lbp_3D_m2_glszm_Small Area Low Gray Level Emphasis,

fusion_lbp_3D_m2_glszm_Zone Entropy,

fusion_log_sigma_3_0_mm_3D_firstorder_10Percentile,

fusion_logarithm_firstorder_Maximum,

fusion_logarithm_glszm_Small Area Emphasis,

fusion_square_firstorder_Median,

fusion_square_glcm_Cluster Shade,

fusion_square_glszm_Large Area High Gray Level Emphasis,

fusion_square_glszm_Small Area Emphasis,

fusion_wavelet_HLL_glszm_Gray Level Variance,

fusion_wavelet_HLL_ngtdm_Complexity,

fusion_wavelet_HLL_ngtdm_Contrast,

fusion_wavelet_LHH_glszm_Small Area High Gray Level Emphasis,

fusion_wavelet_LHL_firstorder_Maximum,

fusion_wavelet_LLH_firstorder_10Percentile,

fusion_wavelet_LLH_firstorder_Maximum,

fusion_wavelet_LLH_firstorder_Median,

fusion_wavelet_LLH_glszm_ZoneVariance

**Fig. S1** Graph of output feature distribution of each model in the training set, internal test set, and external validation set.

**INTRA**

A B


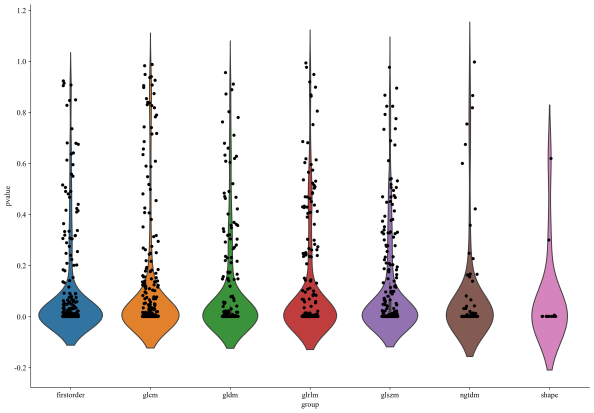

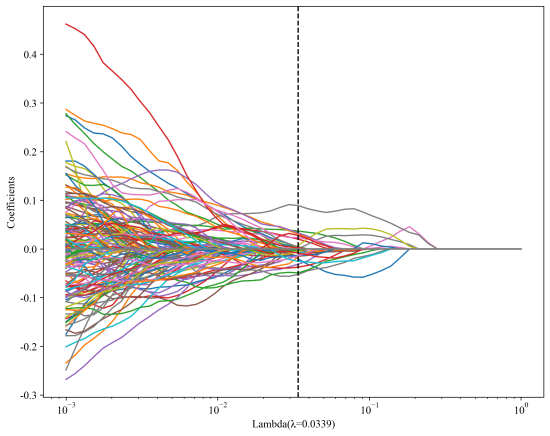


C D


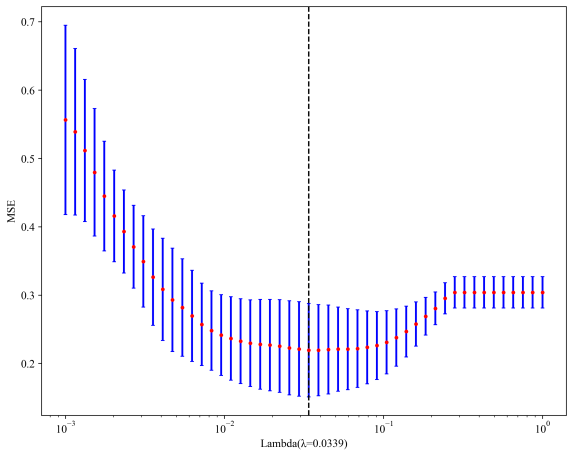

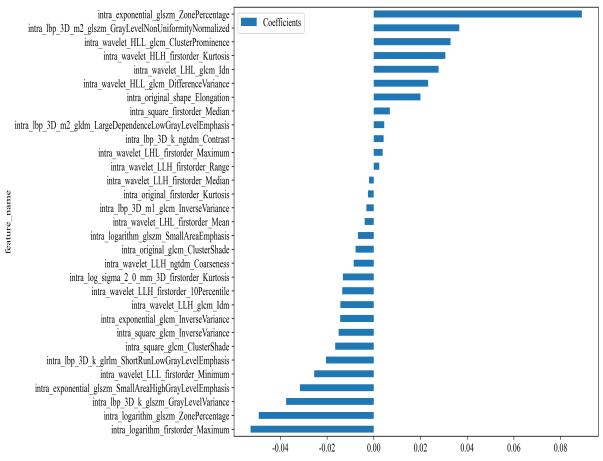


**Peri2mm**

**A B**


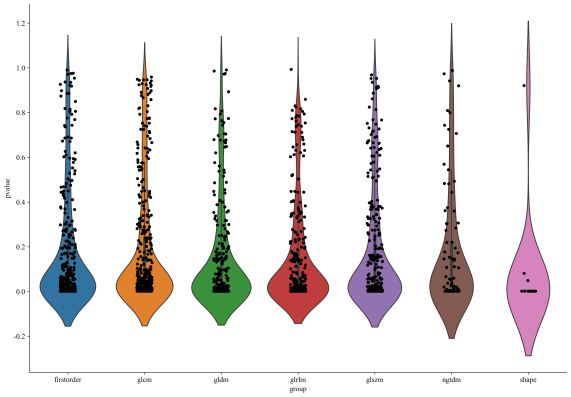

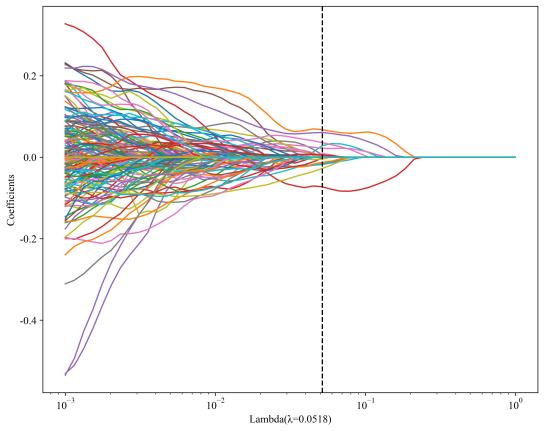


**C D**


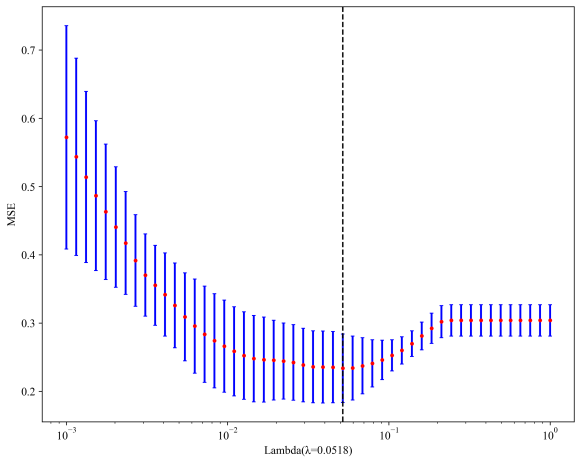

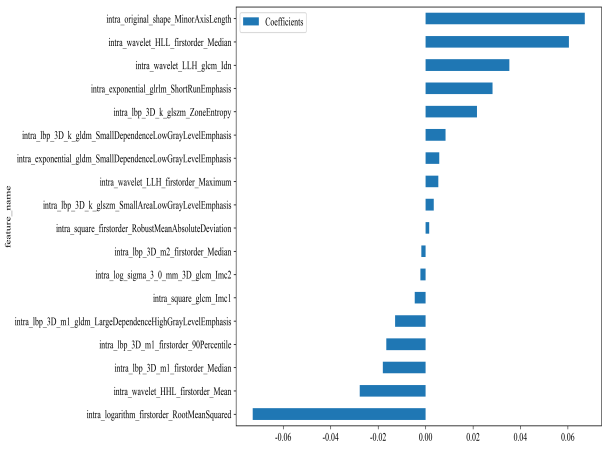


**Fusion2mm**

A B


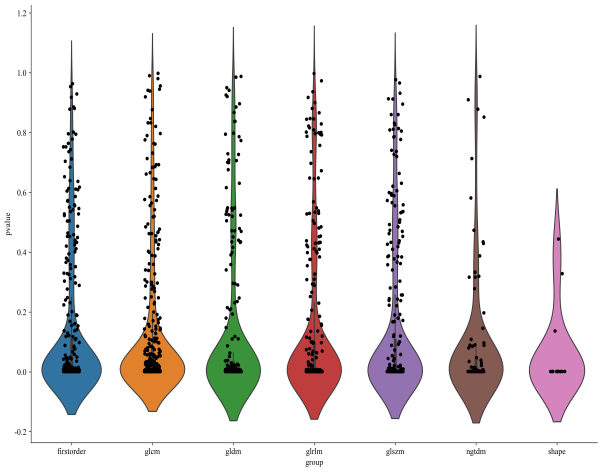

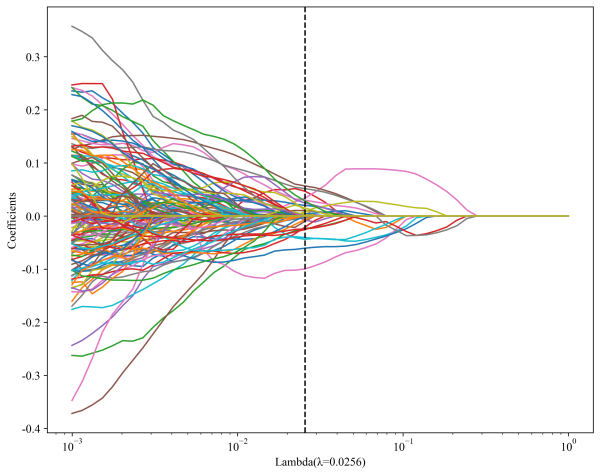


**C D**


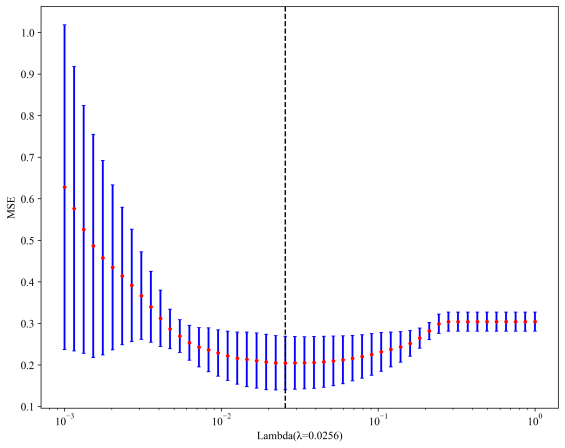

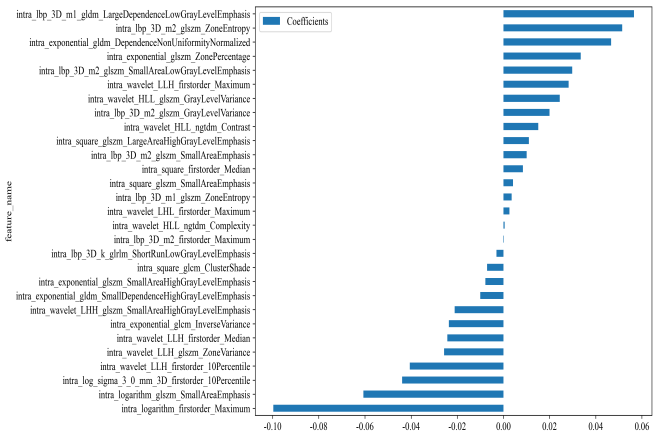


**2D**

**A B**


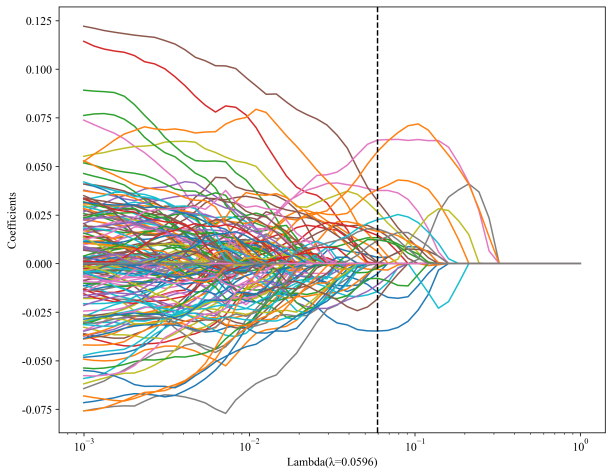

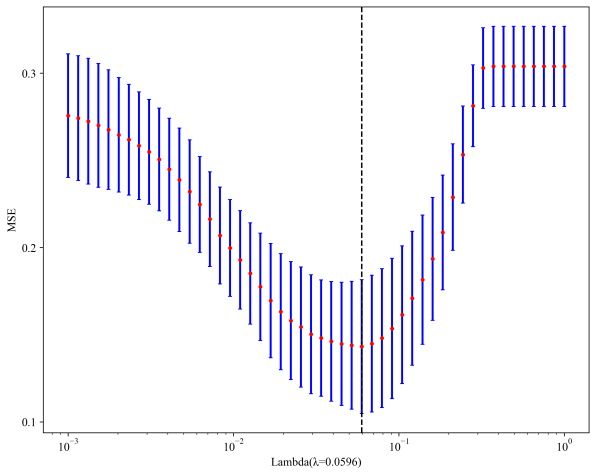


**C**


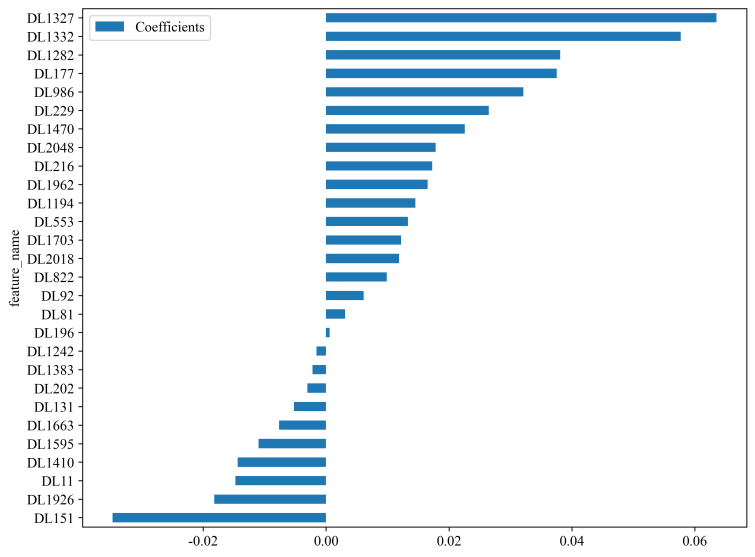


**3D**

**A B**


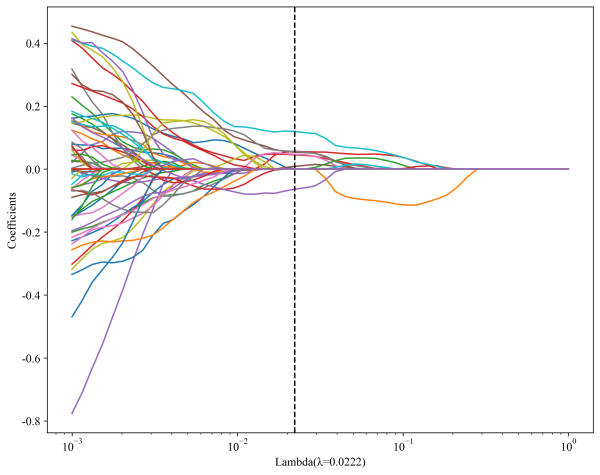

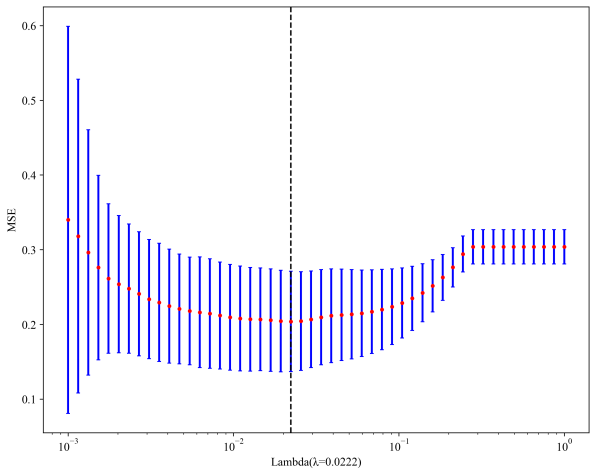


**C**


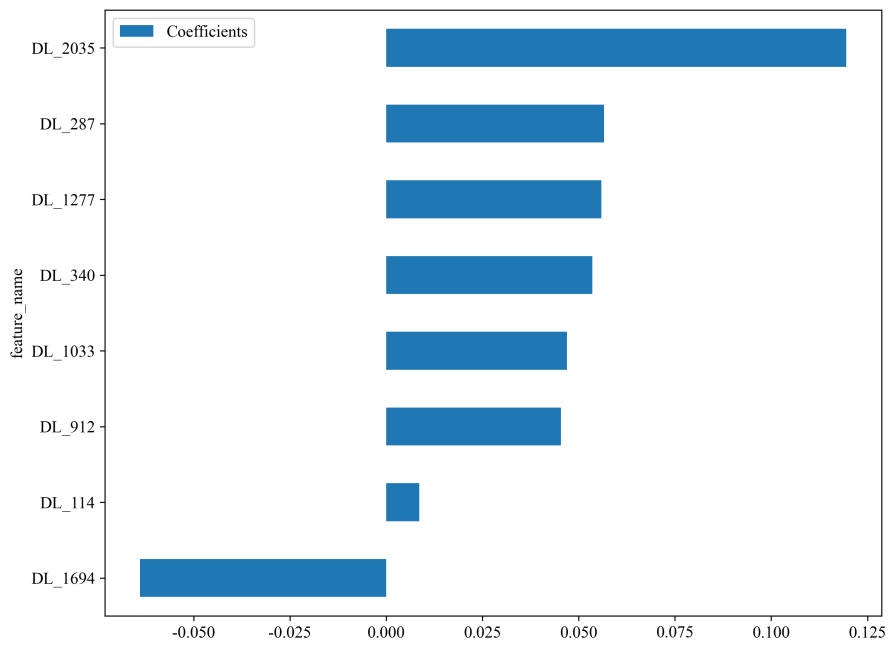


**2.5D**

**A B**


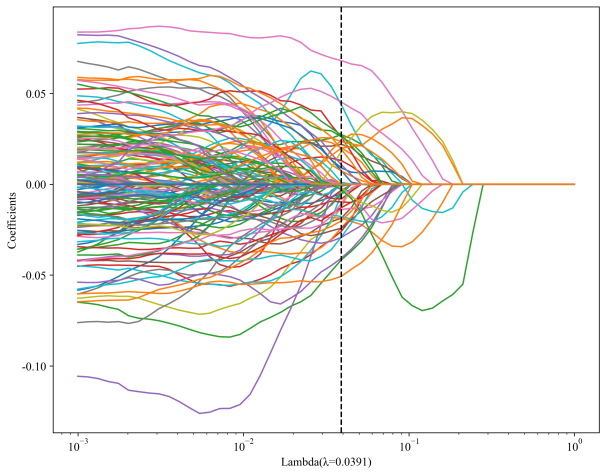

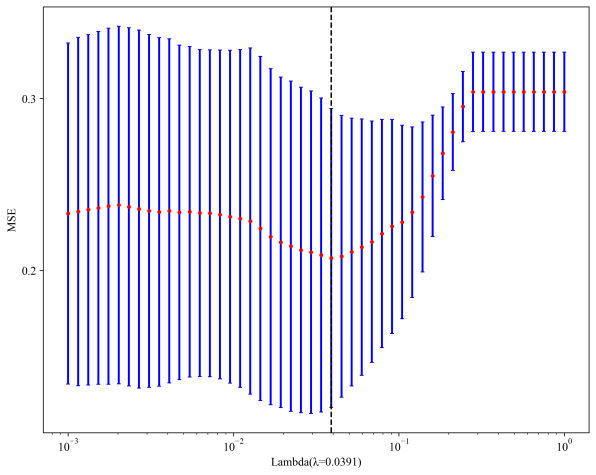


**C**


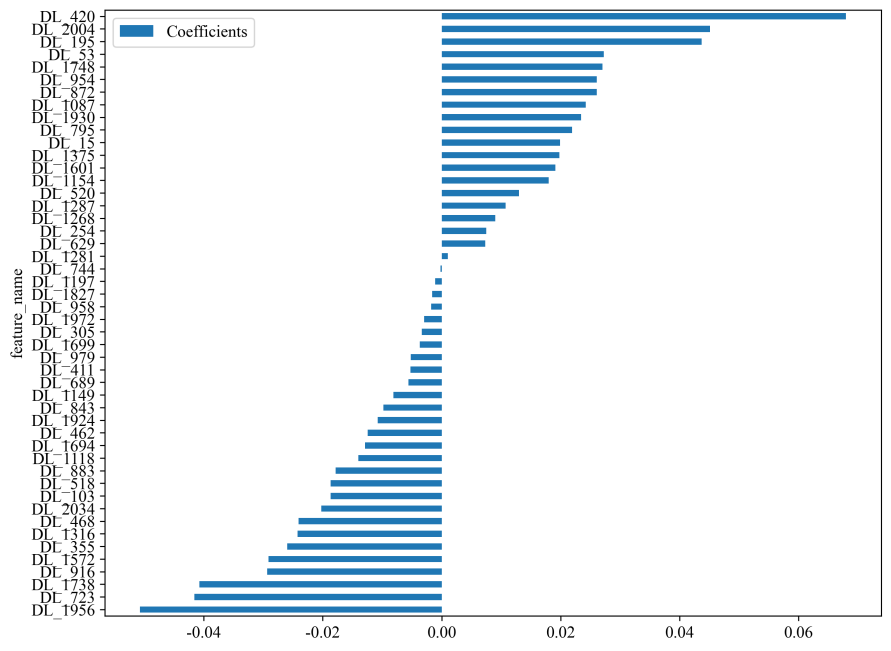


Fig. S2 T-SNE dimension reduction was used for visualization of these features.

**INTRA Peri2mm**


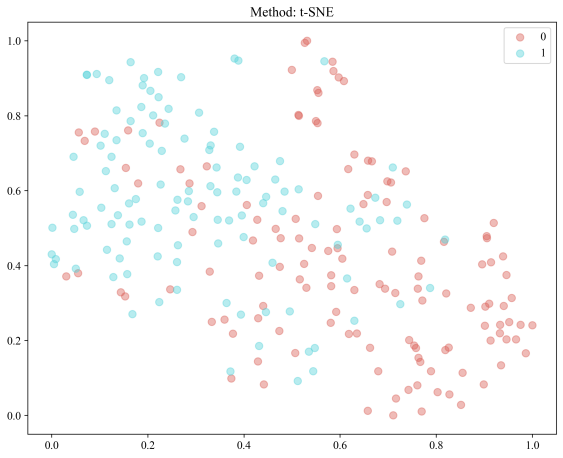

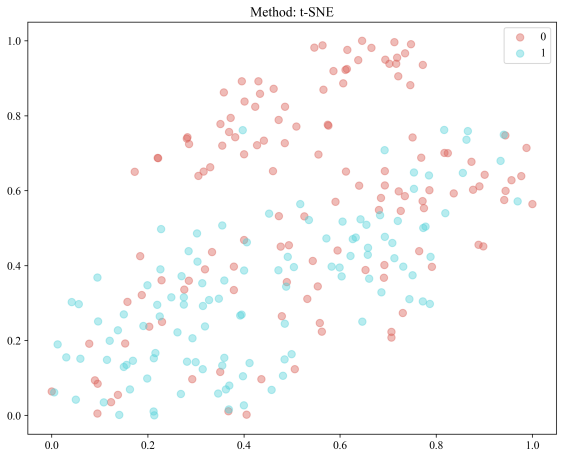


**Fusion2mm 2D**


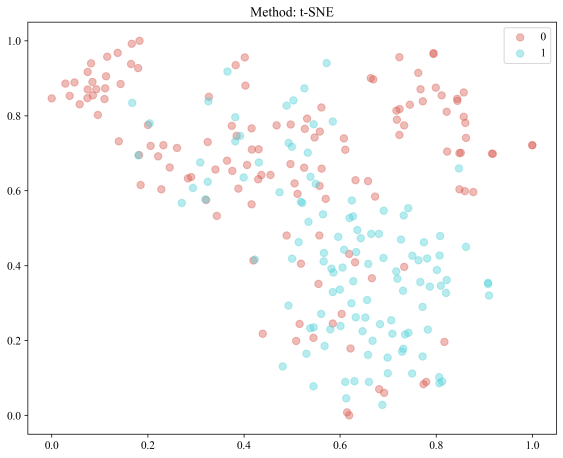

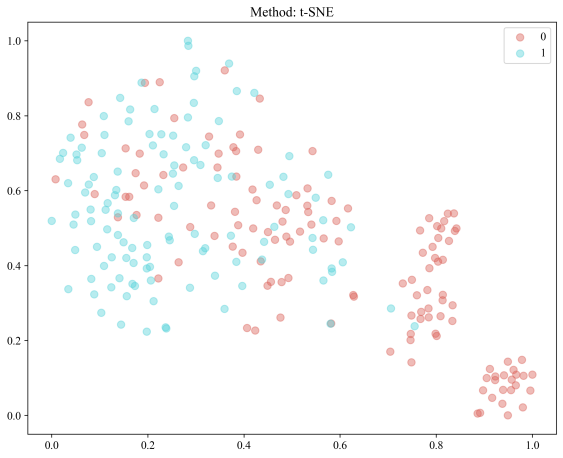


**3D 2.5D**


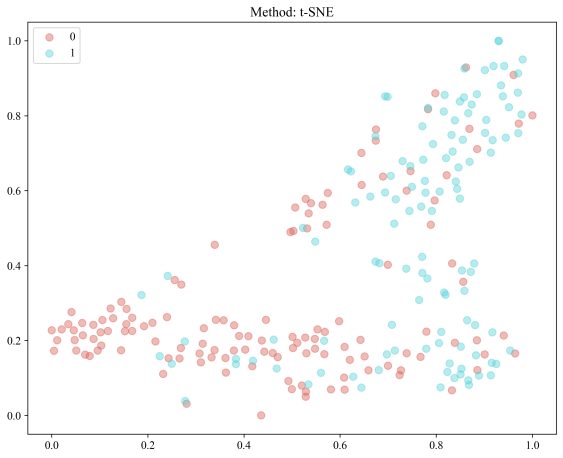

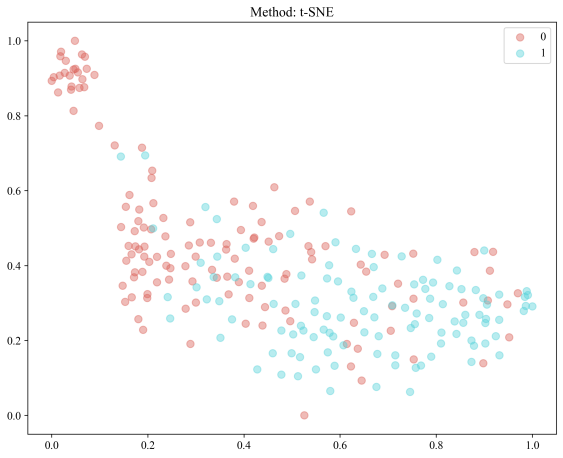


**Fig. S3** Sample prediction confusion matrix of each model in the training set, internal test set, and external validation set.

**INTRA**


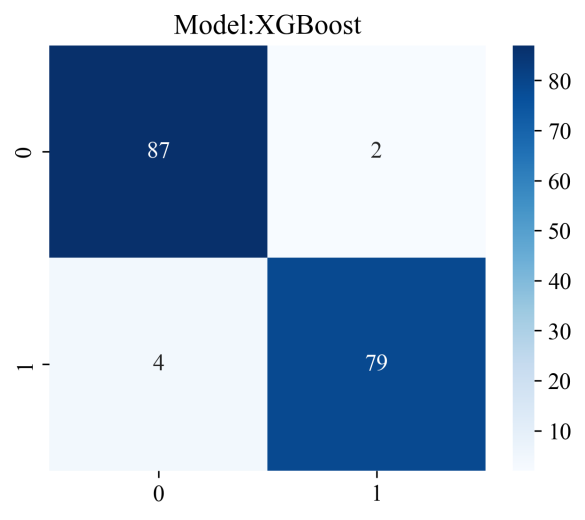

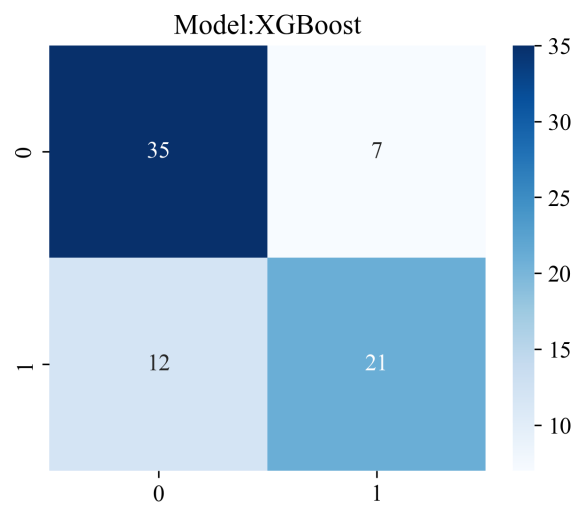


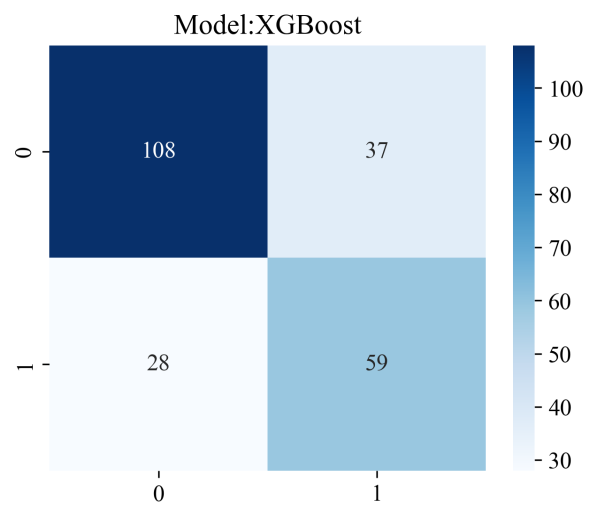


**Peri2mm**


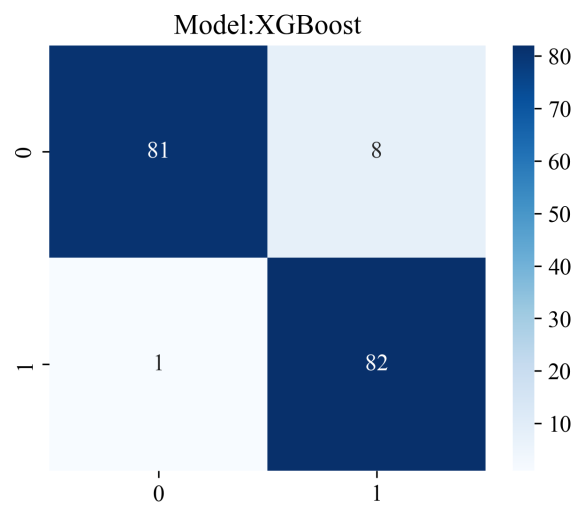

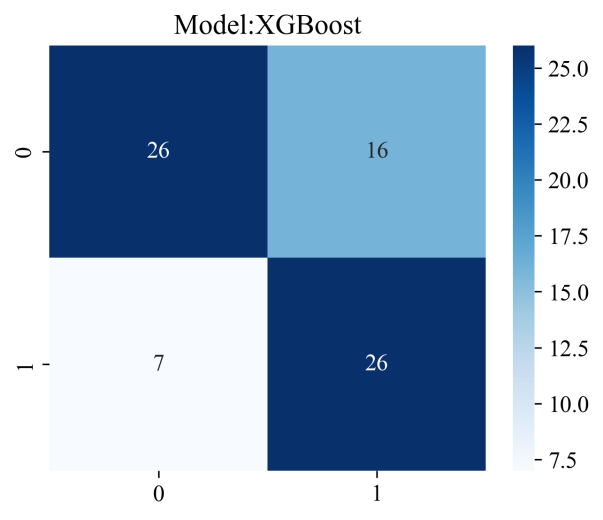


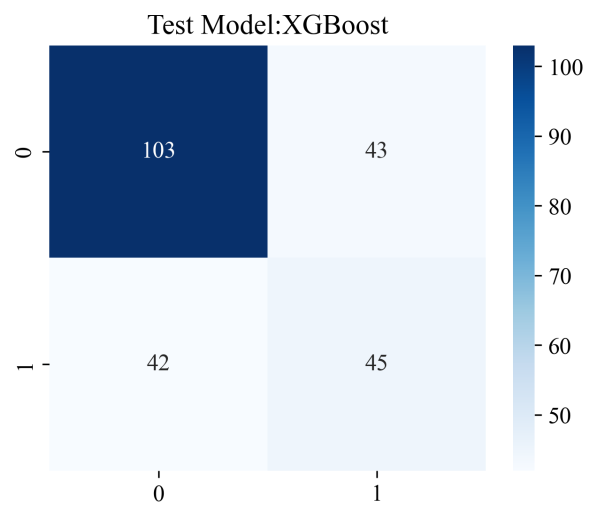


**Fusion2mm**


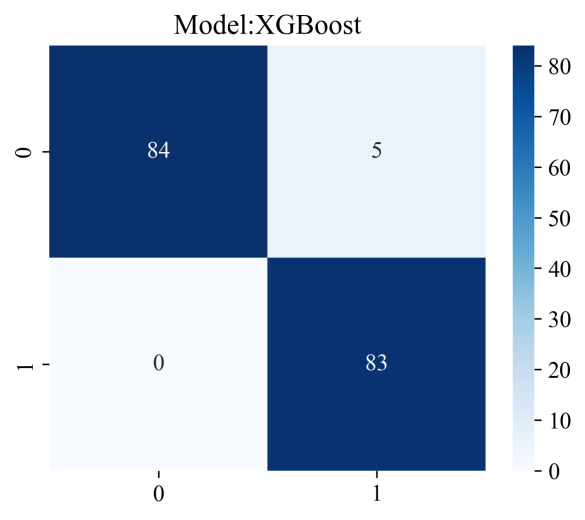

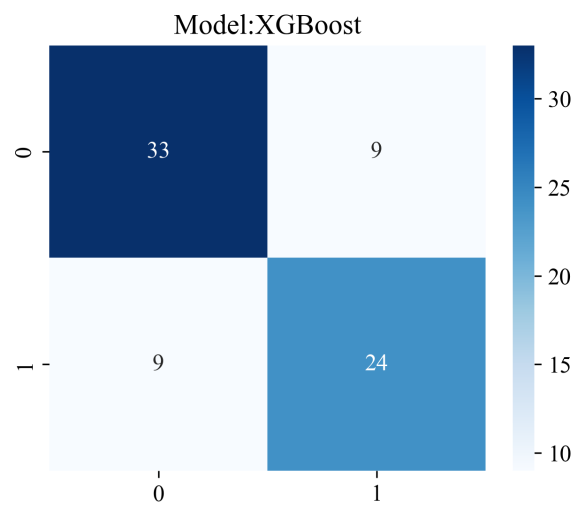


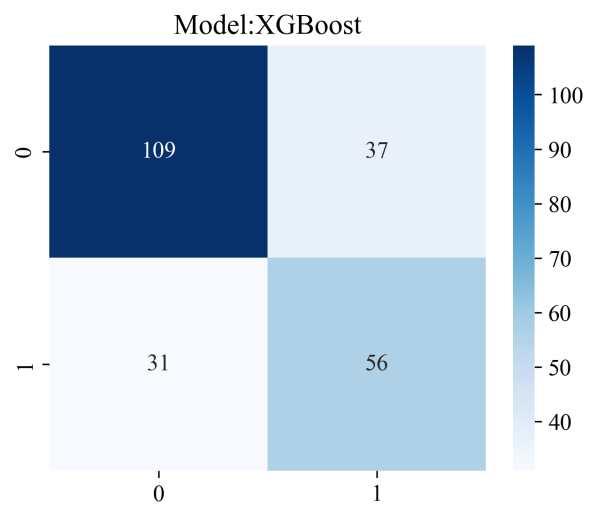


**2D**


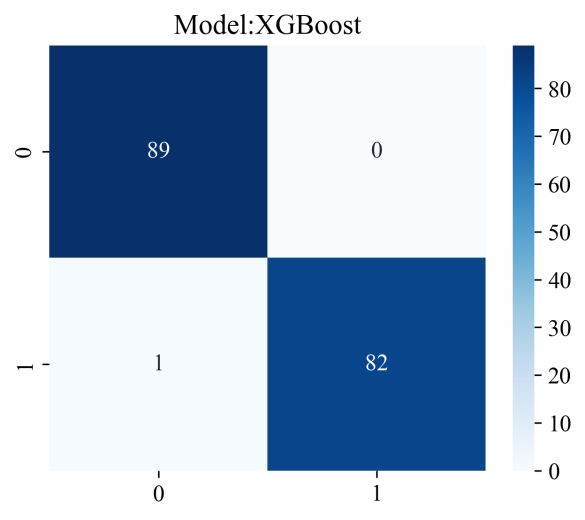

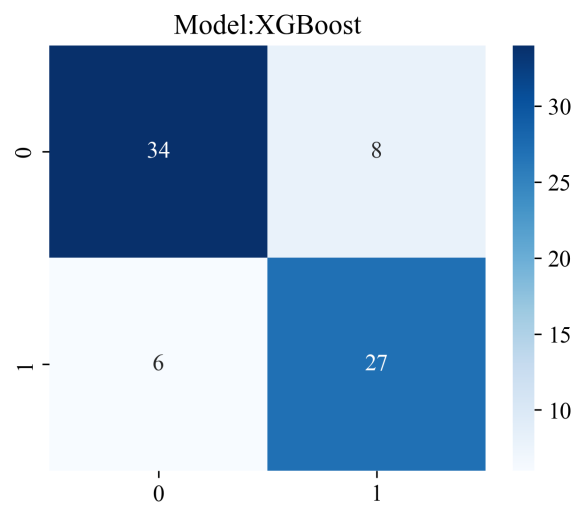


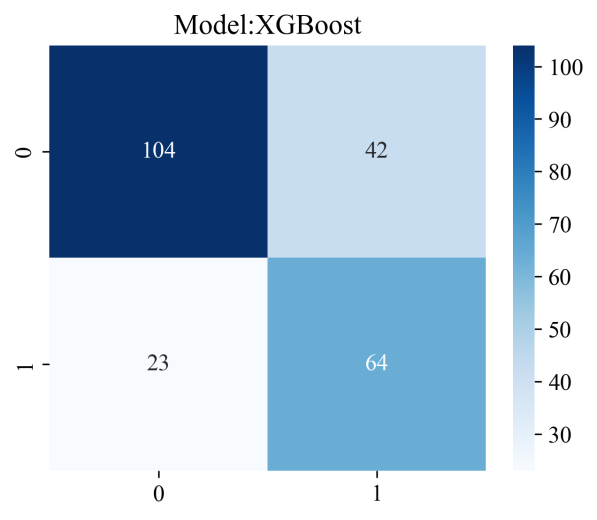


**3D**


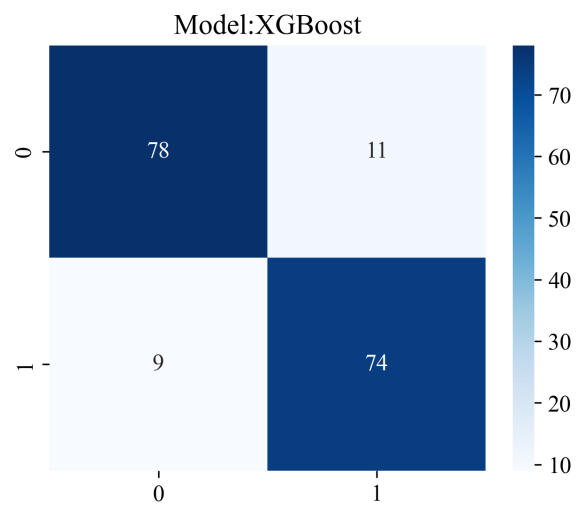

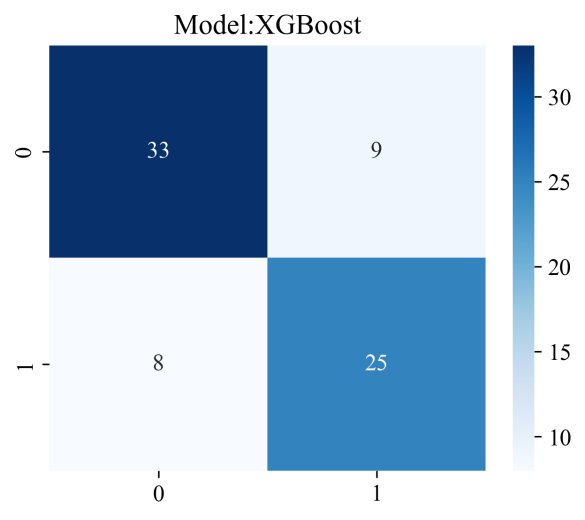


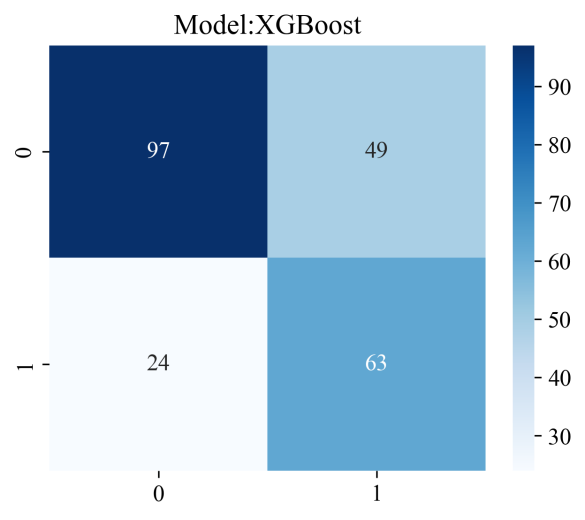


**2.5D**


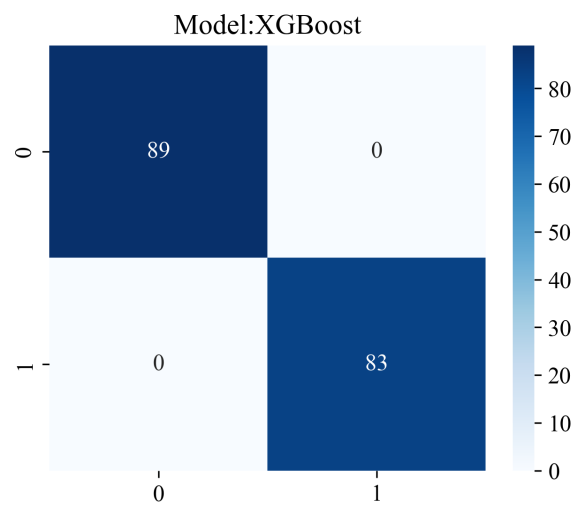

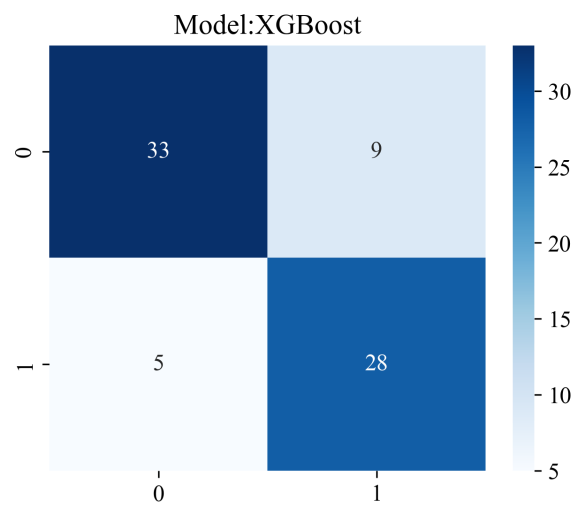


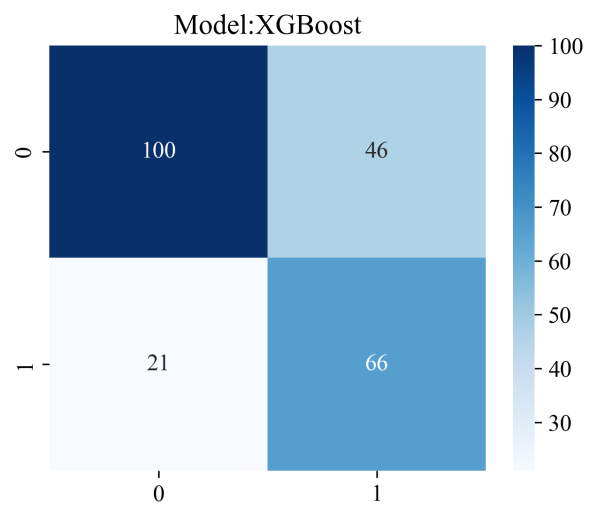


**Fig. S4** Sample prediction histogram of each model in the training set, internal test set, and external validation set.

**INTRA**


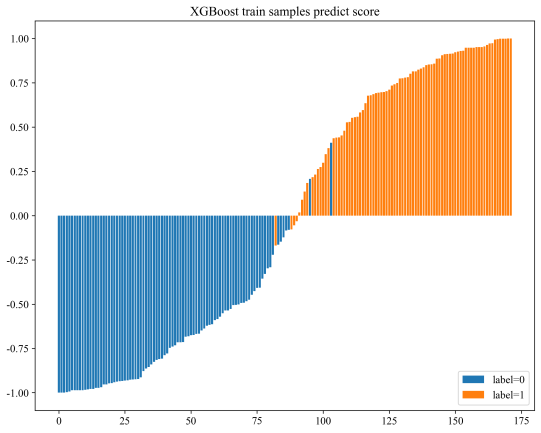

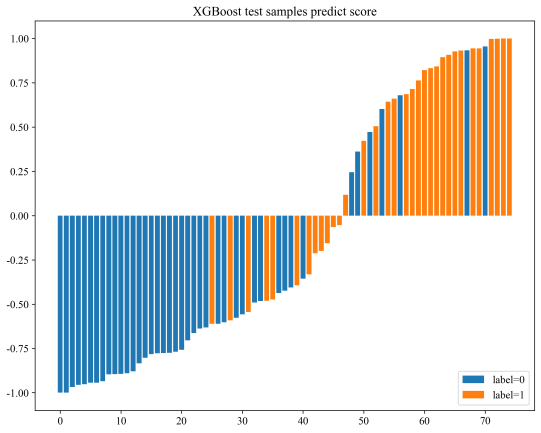


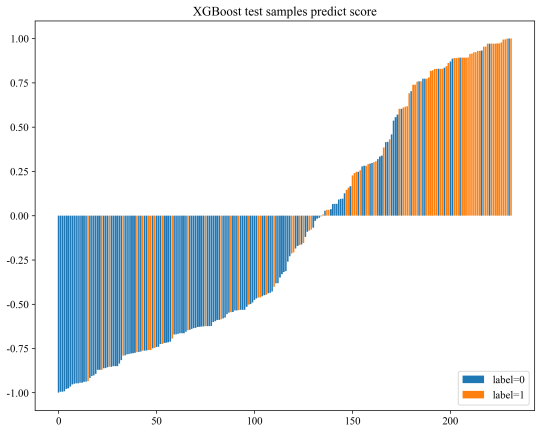


**Peri2mm**


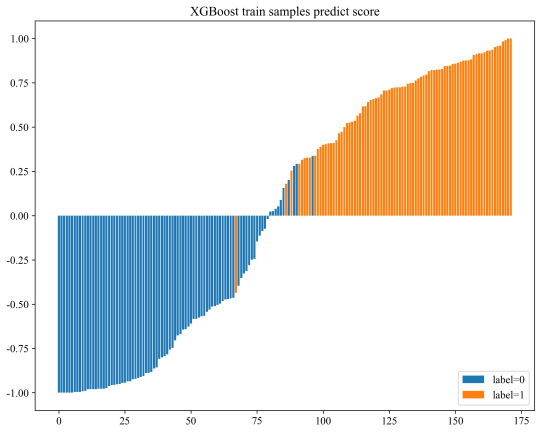

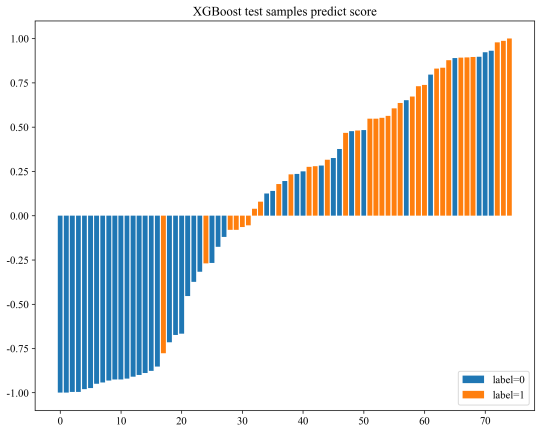


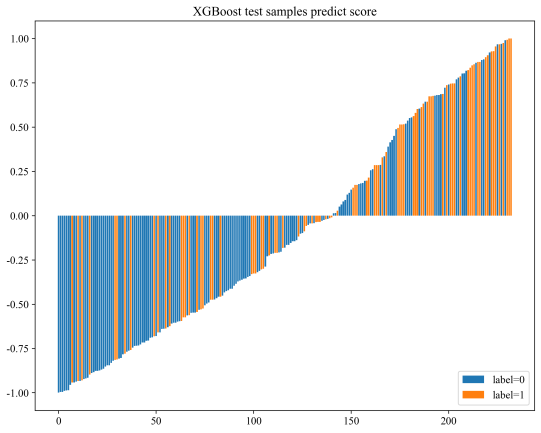


**Fusion2mm**


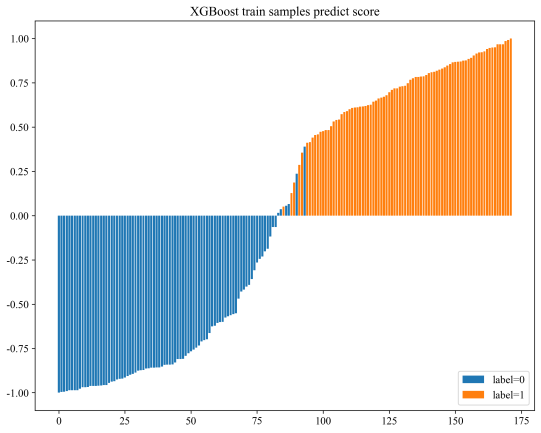

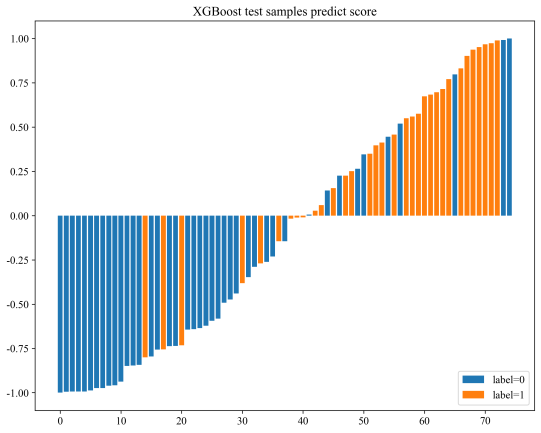


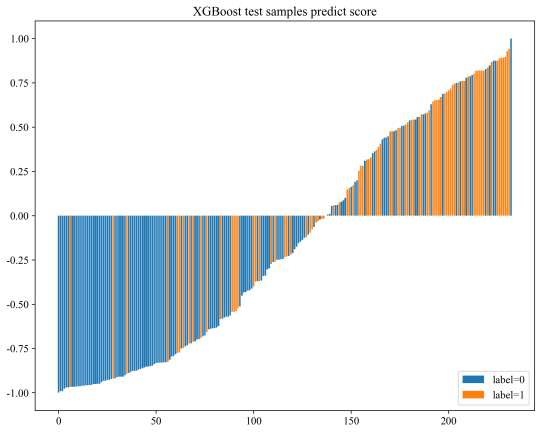


**2D**


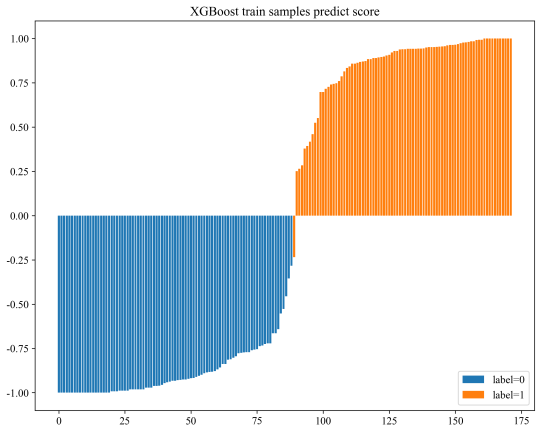

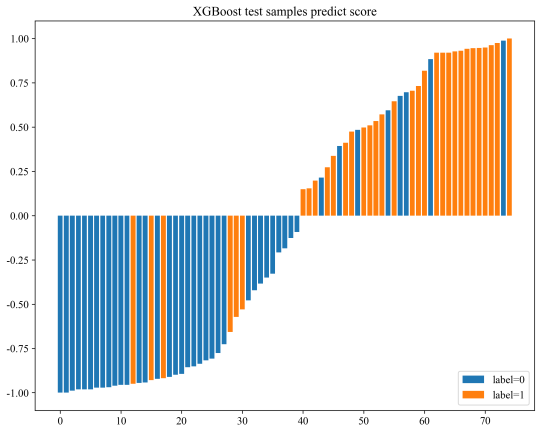


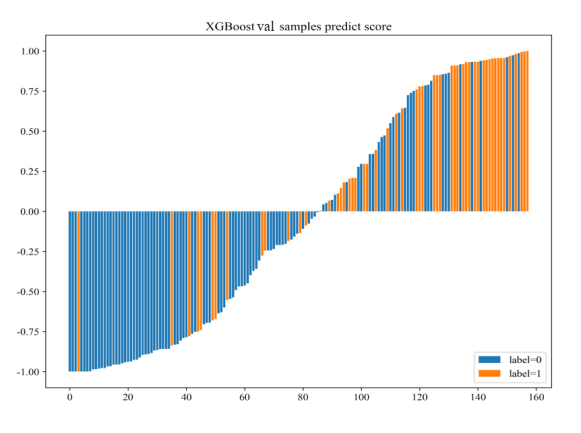


**3D**


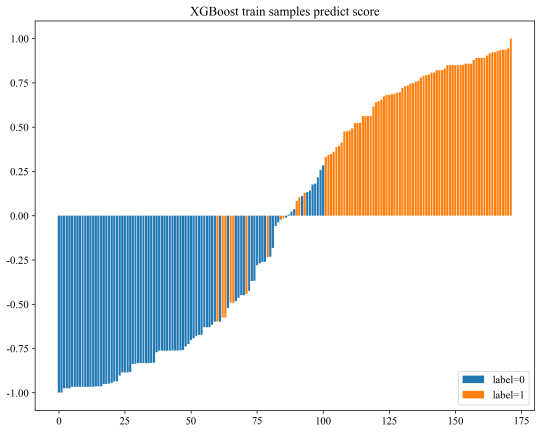

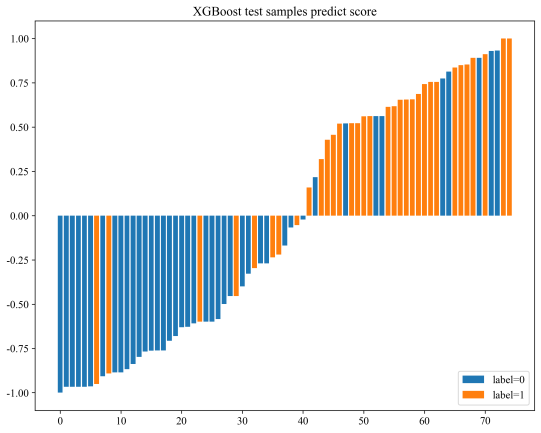


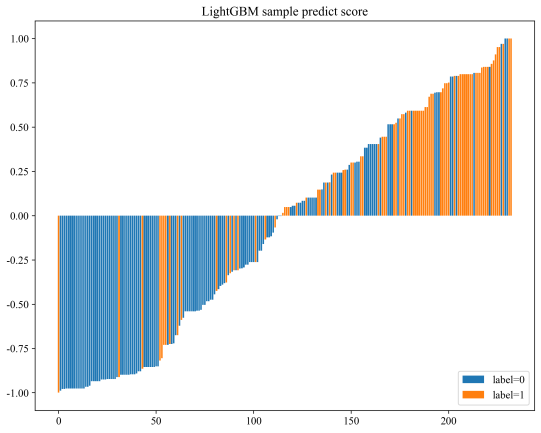


**2.5D**


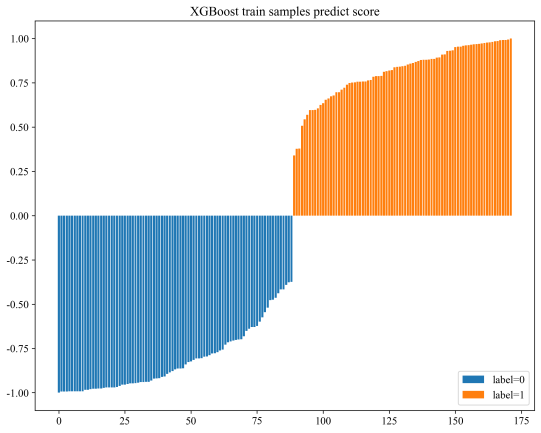

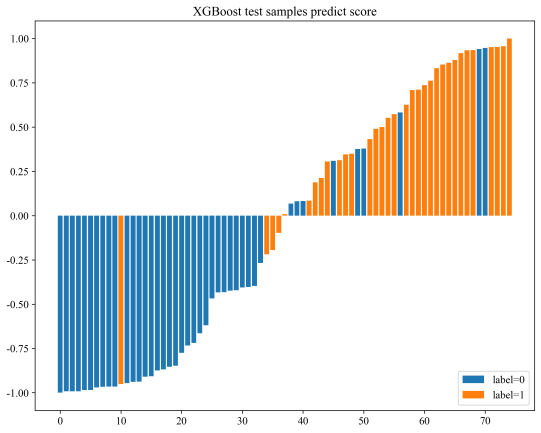


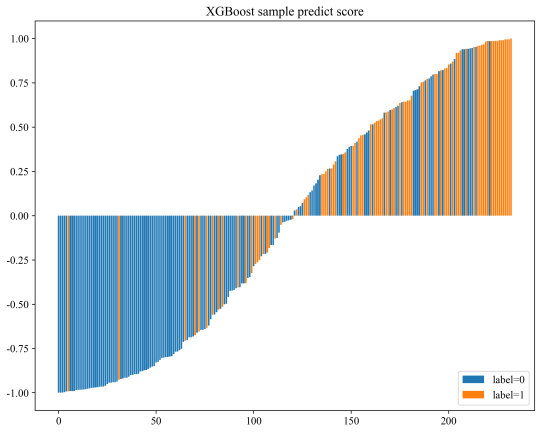

Supplement: Supplementary file 1 [file DataSheet1.docx]
